# Outer Membrane Vesicles as a Versatile Platform for Vaccine Development: Engineering Strategies, Applications and Challenges

**DOI:** 10.1002/jev2.70150

**Published:** 2025-09-08

**Authors:** Asja Garling, Frédéric Auvray, Mathieu Epardaud, Éric Oswald, Priscilla Branchu

**Affiliations:** ^1^ IRSD, Université de Toulouse, INSERM, INRAE, ENVT Toulouse France; ^2^ INRAE, Université de Tours, ISP Nouzilly France; ^3^ CHU de Toulouse, Hôpital Purpan Toulouse France

**Keywords:** antigens, bioengineering, Gram‐negative bacteria, lipopolysaccharides (LPS), outer membrane vesicles (OMVs), Vaccine platform

## Abstract

Outer membrane vesicles (OMVs) are nanosized vesicles naturally secreted by Gram‐negative bacteria and represent a promising platform for vaccine development. OMVs possess inherent immunostimulatory properties due to the presence of pathogen‐associated molecular patterns (PAMPs), providing self‐adjuvanting capabilities and the ability to elicit both innate and adaptive immune responses. This review outlines the advantages of OMVs over traditional vaccine strategies, including their safety, modularity, and the potential for genetic engineering to enable targeted antigen delivery. We describe approaches to enhance OMVs yield and immunogenicity, such as modifications to reduce lipopolysaccharide (LPS) toxicity and systems enabling antigen localization—either on the surface or within the lumen—using fusion constructs like ClyA, Lpp‐OmpA, AIDA‐I, Hbp, and Sec/Tat signal peptides. We further summarize preclinical applications of OMVs‐based vaccines targeting bacterial pathogens, viral infections, and cancer. In addition, we address key challenges in large‐scale production, purification, and long‐term stability, and explore strategies for conjugating or encapsulating heterologous antigens. Overall, OMVs offer a versatile and scalable extracellular vesicle‐based platform with strong potential for next‐generation vaccines targeting diverse infectious diseases and beyond.

## 1 Diversity of Extracellular Vesicles in Gram‐Negative Bacteria

Extracellular vesicles (EVs) are produced by species belonging to the three domains of life (Deatherage and Cookson 2012). These vesicles are involved in intercellular communication, survival and cell protection within stressful environments and during pathogenesis with the transport of toxins (Deatherage and Cookson 2012; Gill et al. 2018). In Prokaryotes, especially in Gram‐negative bacteria, EVs were first observed in 1965 by Bishop and Work (Bishop and Work 1965). They are mainly produced by the budding of the outer membrane (OM), giving them the specific name of Outer Membrane Vesicles (OMVs). They have been widely studied since their discovery, and various roles have been assigned to them. One of them is the export of waste material such as proteins or peptidoglycan fragments originating from cell wall turnover. These molecules accumulate in the periplasm, leading to an increase of osmotic pressure favouring vesiculation (Baeza et al. 2021; McBroom and Kuehn 2007). OMVs have also a critical role in pathogenesis, notably due to their capacity to remotely deliver toxins to the host (Horstman and Kuehn 2000; Kolling and Matthews 1999; Murase et al. 2016). They also have a role in the acquisition of nutrients such as iron upon inflammation (Gasperini et al. 2017). In addition, as their composition is similar to that of the bacterial OM, with the presence of lipopolysaccharide (LPS), phospholipids (PLs) and outer membrane proteins (OMPs), they act as decoys to neutralize phages or deleterious molecules such as host defensins or OM targeting antibiotics (Manning and Kuehn 2011).

Other types of EVs are produced by Gram‐negative bacteria which differ from OMVs in their formation process and composition. Outer‐Inner Membrane Vesicles (O‐IMVs) were first described in 2013 in *Shewanella vesiculosa* M7^T^ (Pérez‐Cruz et al. 2013). It was proposed that O‐IMVs are produced from a protrusion of the inner membrane (IM) through the peptidoglycan layer and the budding of the OM (Pérez‐Cruz et al. 2013). As a result, O‐IMVs contain both cytoplasmic and periplasmic components and may carry nucleic acids such as plasmid DNA. These vesicles have the distinct feature of a double membrane visible under transmission electron microscopy. The production of O‐IMVs has also been described in other Gram‐negative pathogenic bacteria such as *Neisseria gonorrhoeae*, *Pseudomonas aeruginosa* PAO1 and *Acinetobacter baumannii* AB41, where they represented a low proportion of total EVs produced, that is, from 0.1% to 1.2% (Pérez‐Cruz et al. 2013, 2015).

Explosive vesicles, also referred to as Explosive Outer Membrane Vesicles (EOMVs), represent another type of EVs from Gram‐negative bacteria (Toyofuku et al. 2019). Their production was first reported in 2016 (Turnbull et al. 2016). A change from rod‐shaped to round cells in *P. aeruginosa* culture mediated by a prophage‐derived endolysin was observed, resulting in bacterial lysis and the formation of EOMVs (Turnbull et al. 2016). They are usually produced upon environmental stress. In these conditions, bacteria activate their SOS response to survive. However, when this response is not sufficient, they undergo bacterial lysis. In this case, cytoplasmic components such as proteins and DNA as well as periplasmic proteins and peptidoglycan are released and surrounded by membrane fragments to form explosive vesicles. The proportion of EOMVs produced compared to other types of EVs remains unknown. However, EOMVs seem to be a minority since less than 1% of the bacterial population of *P. aeruginosa* undergoes the SOS response in the absence of stress conditions (Turnbull et al. 2016).

Among these different types of EVs produced by Gram‐negative bacteria, OMVs have received an increased attention in the past decade, as these nanoparticles can be used as a vaccine platform to fight against microbial pathogens. Indeed, their immunogenicity, intrinsic adjuvant properties and their inability to replicate are the major advantages for using OMVs as a vaccine platform over attenuated or inactivated bacteria (Bielaszewska et al. 2018; Kaparakis‐Liaskos and Ferrero 2015; Mancini et al. 2020). Various engineering techniques have been developed to obtain OMVs with the required properties (Balhuizen et al. 2021; Micoli and MacLennan 2020).

In this review, we will focus on OMVs produced by Gram‐negative bacteria and the advantages of their use for the development of a vaccine platform. Examples of applications since their discovery will be provided. Since the location of antigens is crucial for eliciting an effective immune response against pathogens, various systems designed to present specific antigens in OMVs fraction will be described. We will discuss the challenges associated with large‐scale OMVs production, including purification step, safety concerns and long‐term stability. Additionally, we will provide a comprehensive overview of the key parameters involved in optimizing OMVs production, including the selection of the most suitable bacterial strain for the development of OMV‐based vaccines. Finally, we will explore future directions for OMVs vaccines, including their potential applications against emerging pathogens and in cancer immunotherapy.

## 2 Structure and Composition of OMVs

**FIGURE 1:**
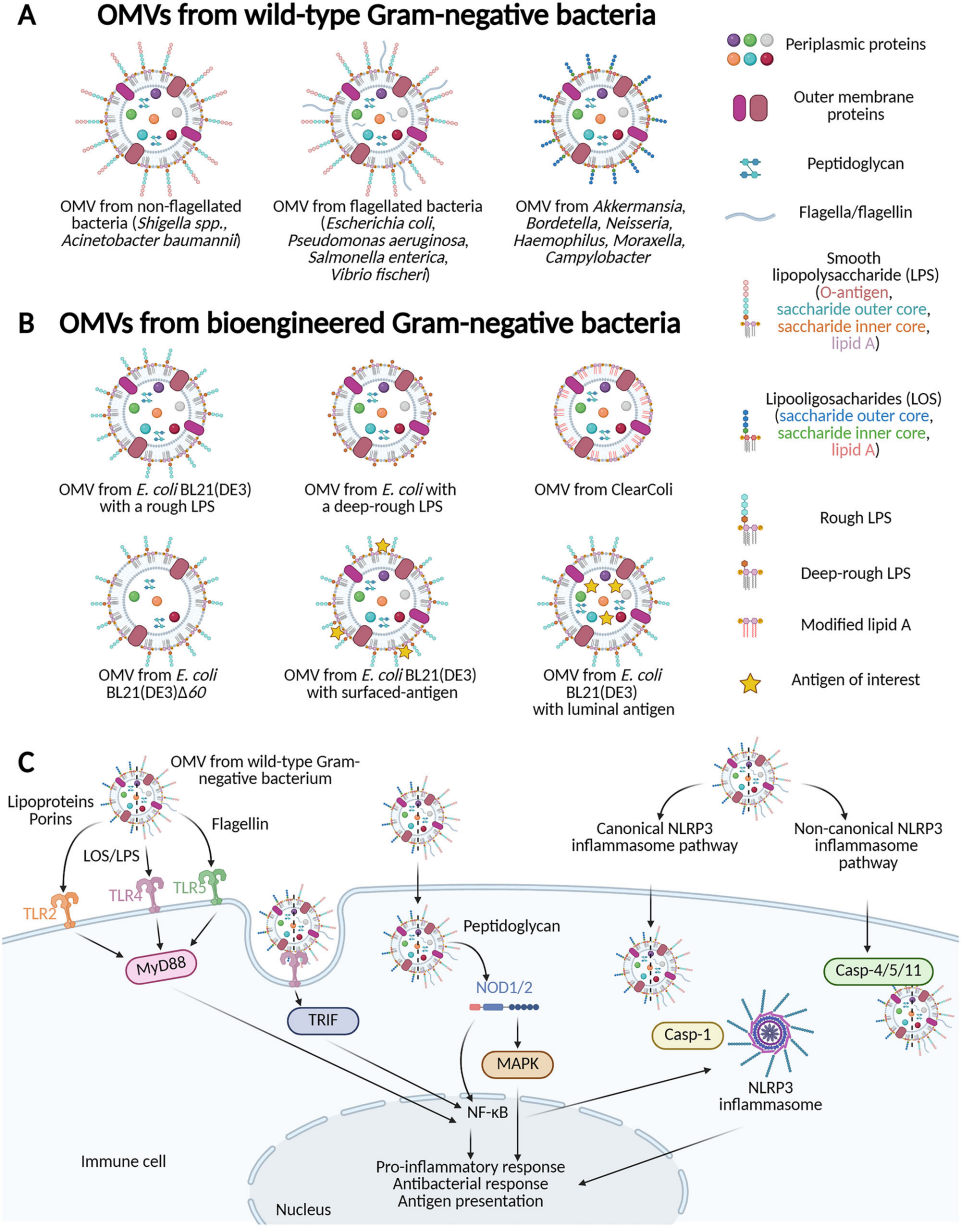
The different types of Outer Membrane Vesicles (OMVs) with their immunogenic and adjuvant properties. (A) OMVs from various wild‐type (WT) Gram‐negative bacteria, including flagellated and non‐flagellated bacteria with smooth lipopolysaccharides (LPS) (left and middle) or from bacteria with lipooligosaccharides (LOS) (right). (B) OMVs from bioengineered Gram‐negative bacteria. OMVs‐producing bacteria could be bioengineered to express rough or deep‐rough LPS, leading to the reduction of the immunogenicity of LPS such as in *Escherichia coli* BL21(DE3) strain. Bioengineering could be also used to modify lipid A and reduce the toxicity of LPS such as in ClearColi strain. In addition, the mutation of genes encoding membrane or periplasmic proteins could favour the immunogenicity of the antigens of interest such as in the *E. coli* BL21(DE3)Δ*60* strain. Finally, antigens of interest could be added either to the surface of OMVs or in their lumen. C) OMVs recognition by immune cells and activation of inflammatory signalling pathways. The recognition of OMVs‐associated pathogen‐associated molecular patterns (PAMPs) and their trafficking into the immune cells trigger different signalling pathways. Indeed, lipid A of OMVs from WT Gram‐negative bacteria is recognized by Toll‐like receptor 4 (TLR4) of immune cells, inducing MyD88‐ and Toll/IL‐1R domain‐containing adaptor‐inducing IFN‐β (TRIF)‐dependent pathways and leading to the activation of the nuclear factor kappa B (NF‐κB). Lipoproteins and flagellin present on the surface of OMVs are recognized by TLR2 and TLR5, respectively, inducing the MyD88‐dependent pathway. Peptidoglycan in the lumen of OMVs is recognized by NOD1/2 cytosolic receptors inducing NF‐κB and mitogen‐activated protein kinase (MAPK) signalling cascade. NF‐κB is also implicated in the formation and activation of the NOD‐like receptor family pyrin domain‐containing protein 3 (NLRP3) inflammasome. The canonical pathway of the NLRP3 inflammasome, involving the caspase‐1, is activated following the detection of stress by the immune cell caused by the presence of OMVs. Besides, the non‐canonical NLRP3 inflammasome pathway is activated by the recognition of intracellular LPS of OMVs by caspase‐4 (casp‐4) and caspase‐5 (casp‐5) (or caspase‐11 in mice). All of these pathways both lead to proinflammatory and antibacterial responses and antigen presentation. Created with Biorender.

OMVs are spherical nanoparticles produced from the budding of the OM of Gram‐negative bacteria. Their size ranges from 20 to 250 nm (Qing et al. 2019; Schwechheimer and Kuehn 2015). The bacterial envelope of Gram‐negative bacteria includes two membranes: the IM, containing mainly PLs and transmembrane proteins, and the OM containing mostly PLs, LPS and proteins, including OMPs. These two membranes are separated by a periplasmic space containing a thin layer of peptidoglycan (Bos and Tommassen 2004; Schwechheimer and Kuehn 2015). The bacterial OM has an asymmetrical structure with PLs in the inner leaflet, facing the periplasm, while the LPS and surface‐exposed proteins (such as pili or fimbriae) are located in the outer leaflet. Different types of LPS have been described according to their structure. The smooth LPS is composed of three regions: (i) the O‐antigen is a repetitive glycan polymer at the extremity of the LPS and the most variable part across bacterial strains, thus defining their serogroup. O‐antigen has strong antigenic properties and represents the major target of host antibodies during an infection; (ii) the core oligosaccharide contains an inner core composed of both keto‐deoxyoctulosonate (Kdo) and heptose, while its outer core is composed of glucose, mannose, galactose and heptose residues and (iii) the lipid A, also known as the endotoxin, composed of a dimer of phosphorylated glucosamine attached to fatty acid chains, which is the hydrophobic part of LPS anchored in the OM (García‐Weber and Arrieumerlou 2021). An LPS molecule lacking the O‐antigen is called ‘rough LPS’ while an LPS lacking the O‐antigen, the saccharide outer core and the total or partial part of the saccharide inner core is called ‘deep‐rough LPS’ (García‐Weber and Arrieumerlou 2021) (Figure 1B).

Some bacterial species lack the O‐antigen and instead express lipooligosaccharides (LOS). These species include mucosal commensals such as *Akkermansia muciniphila* (Garcia‐Vello et al. 2024), mucosal pathogens from the genera *Bordetella*, *Neisseria*, *Haemophilus* and *Moraxella*, as well as enteric pathogens like *Campylobacter* (Preston et al. 1996) (Figure 1A). LOS are the major glycolipids of the OM and their composition resembles that of rough LPS. However, while the absence of the O‐antigen in rough LPS results from mutations in enzymes involved in the LPS biosynthesis pathway (Yethon et al. 1998), LOS are synthesized via a distinct pathway and naturally lack this polysaccharide component (Preston et al. 1996; Arking et al. 2001). Compared to rough LPS, LOS typically contain shorter, non‐repeating oligosaccharides in their core region, usually comprising fewer than ten sugar residues (Mandrell and Apicella 1993). Moreover, LOS differ from rough LPS in their structural and antigenic resemblance to human glycolipids (Preston et al. 1996; Harvey et al. 2001). Importantly, LOS can undergo structural modification both *in vitro* and *in vivo* through the addition of sialic acid to the terminal galactose in its lactosamine structure. This is achieved using the host‐derived compound cytidine 5'‐monophospho‐N‐acetylneuraminic acid (CMP‐NANA) (Gibson et al. 1993; Mandrell and Apicella 1993). Such modification masks the LOS from host immune recognition, rendering the bacteria resistant to complement‐mediated serum killing and enabling immune evasion (Nairn et al. 1988; Mandrell et al. 1990; Gibson et al. 1993).

The protein and lipid composition of the OMVs membrane is similar to that of the bacterial OM described above, with LPS, LOS, fimbriae and pili on the outer leaflet, PLs principally within the inner leaflet, and OMPs and lipoproteins embedded in the lipid bilayer. Moreover, flagellin, the structural component of the flagellar filament, can be enriched in OMVs extracts from flagellated bacteria (i.e., *Escherichia coli*, *Salmonella enterica*, *P. aeruginosa* or *Vibrio fischeri*) (Bauman and Kuehn 2006; Manabe et al. 2013; Altindis et al. 2014; Aschtgen et al. 2016; Liu et al. 2016; Blackburn et al. 2021) (Figure 1A). However, the location of flagellin in OMVs fractions has not been clarified. The lumen of OMVs contains periplasmic proteins and peptidoglycan fragments (Schwechheimer et al. 2013). However, slight differences in protein, lipid and LPS composition have been identified between OMVs and the bacterial OM (Zingl et al. 2021). For example, OmpX was found to be enriched in OMVs compared to OM samples from the enterotoxigenic *E. coli* ETEC‐2 strain. Conversely, OmpW was more abundant in the bacterial OM than in OMVs (Horstman and Kuehn 2000).

It is noteworthy that, in contrast to O‐IMVs and EOMVs, OMVs are devoid of cytoplasmic compounds such as DNA, RNA or cytosolic proteins (Toyofuku et al. 2023). However, due to the recent discovery of various types of vesicles, some studies appear to conflate these types, by falsely referring to OMVs to describe vesicles that contain cytoplasmic elements (Qing et al. 2019; Mancini et al. 2020; Dhurve et al. 2022; Sirisaengtaksin et al. 2023). The nature of EVs can vary depending on culture conditions and purification methods. We will address the challenges related to the production of OMVs in the Section 7 ‘Challenges associated with increased and large‐scale production, purification, safety and long‐term stability of OMVs’. This unique feature of OMVs—the absence of cytoplasmic components—makes them safer and particularly suitable for vaccine development, as they do not carry genetic material that could potentially encode toxins or facilitate horizontal gene transfer, especially of multidrug resistance genes.

Gram‐negative bacteria rely on different mechanisms to preserve the integrity of their cell envelope. For example, the repulsive charges of LPS help to maintain the OM inflexibility (Adams et al. 2014). In addition, integrity of the bacterial cell wall is supported by some OMPs and lipoproteins (i.e., OMPs with a lipidic moiety directly interacting with the OM lipids) by covalently binding the peptidoglycan to the OM (Bos and Tommassen 2004; Mathelié‐Guinlet et al. 2020), or by a balanced asymmetry in PLs in the OM. The disruption of one of these mechanisms results in the budding of the OM and therefore in the production of OMVs (Roier et al. 2016; Schwechheimer et al. 2013). In addition, the major PLs and the lipid A in the OM have different geometric shapes such as cylindrical, conical and inverted cone revealing microdomains in the OM with negative or positive curvature. These microdomains favour localized budding of the OM and the production of OMVs (Giordano et al. 2020). It is noteworthy that OMVs composition and content could be modulated depending on the mechanism by which they are produced. This will be more detailed in Section 7.1 ‘Mechanisms of OMVs overproduction and bioengineering of bacterial strains to enhance the production of OMVs vaccines’.

## 3 Immunogenic and Adjuvant Properties of OMVs

Adjuvants are compounds that stimulate the immune response and improve the vaccine potency of antigens (Kashyap et al. 2022; Tizard 2021). As mentioned above, OMVs have a composition similar to that of the OM of the parental bacteria (with LPS or LOS, OMPs, lipoproteins, PLs and flagellin from flagellated bacteria), and include periplasmic content in their lumen (Toyofuku et al. 2023). Some of these compounds are classified as pathogen‐associated molecular patterns (PAMPs) with immunogenic properties which provide a self‐adjuvanticity to OMVs (Mancini et al. 2020).

LPS is the major immune stimulator found on the surface of OMVs (Mancini et al. 2020). LPS is recognized by Toll‐like receptor 4 (TLR4) exposed on the surface of some immune cells. This triggers the MyD88‐dependent and the Toll/IL‐1R domain‐containing adaptor‐inducing IFN‐β (TRIF)‐dependent pathways described elsewhere (Ciesielska et al. 2021; Kawasaki and Kawai 2014) (Figure 1C). These pathways lead to the activation of the nuclear factor kappa B (NF‐κB), a key orchestrator of the expression of proinflammatory genes, such as those encoding the tumour necrosis factor α (TNF‐α), interleukin 6 (IL‐6), type I interferon (IFNβ), pro‐IL‐1β and pro‐IL‐18 (Giordano et al. 2020; Kawasaki and Kawai 2014; Pellegrini et al. 2017). NF‐κB is also implicated in the formation and activation of the NOD‐like receptor family pyrin domain‐containing protein 3 (NLRP3) inflammasome (Blevins et al. 2022; Pellegrini et al. 2017). This NLRP3 inflammasome is activated in a variety of cells implicated in the initiation of the immune response such as macrophages, dendritic cells, neutrophils and epithelial cells. It is a protein complex involving NLRP3 protein, the adaptor protein apoptosis‐associated speck‐like protein (ASC) and the pro‐caspase‐1 (Pellegrini et al. 2017). The latter is activated into mature caspase‐1, which is implicated in the canonical pathway of the NLRP3 inflammasome following detection of stress by the immune cell caused by the OMVs (Down et al. 2020; Pellegrini et al. 2017) (Figure 1C). The caspase‐1 cleaves the pro‐IL‐1β and pro‐IL‐18 leading to their maturation (Pellegrini et al. 2017). In addition, the non‐canonical pathway of the NLRP3 inflammasome is activated, due to the sense of intracellular OMVs carrying LPS by caspases 4 and 5 in humans (or caspase‐11 in mice) (Blevins et al. 2022; Down et al. 2020) (Figure 1C). As a result of the activation of the canonical and non‐canonical pathways of the NLRP3 inflammasome, cleavage of the gasdermin D by caspases‐1/4/5/11 leads to pores formation in the cell membrane , triggering the pyroptotic cell death. This also induces the release of IL‐1β and IL‐18 in the extracellular environment to control the immune response (Blevins et al. 2022; Down et al. 2020; Pellegrini et al. 2017).

LOS also confer adjuvant properties to OMVs. Their recognition by TLR4 induces the MyD88‐dependent and TRIF‐dependent pathways (Fathy Mohamed and Fernandez 2024; Garcia‐Vello et al. 2024). As described for OMVs containing LPS, OMVs with LOS also activate the canonical and non‐canonical pathways of the NLRP3 inflammasome (Elizagaray et al. 2020) (Figure 1C).

Besides LPS or LOS, other PAMPs from OMVs are recognized by TLRs expressed on the surface of cells. For example, TLR1/TLR2 recognize triacyl lipoproteins, TLR2/TLR6 recognize diacyl lipoproteins and porins and TLR5 recognizes flagellin (Kumar et al. 2009; Li et al. 2020; Mancini et al. 2020; Zhu et al. 2021). The recognition of these PAMPs induces the MyD88‐dependent pathway, leading to the activation of NF‐κB and the proinflammatory response (Kumar et al. 2009).

Pattern recognition receptors (PRRs) other than TLRs, such as NOD‐like receptors (NLRs), are also involved in the recognition of OMVs‐associated PAMPs. NLRs are receptors localized in the cytosol of host cells (Sundaram et al. 2024). Once OMVs have entered host cells, peptidoglycan present in the lumen of OMVs is recognized by NOD1 and NOD2 (Kaparakis et al. 2010; Wolf and Underhill 2018). As a result, two signalling cascades are induced, through NF‐κB and the mitogen‐activated protein kinase (MAPK), leading to a proinflammatory response (Wolf and Underhill 2018) (Figure 1C).

Finally, OMVs‐associated PAMPs confer adjuvant properties by triggering multiple PRRs of the host innate immune system, promoting an inflammatory response, with subsequent initiation of adaptive immunity. Indeed, the processing of OMVs‐associated antigens by innate immune cells and their presentation to adaptive effectors favours the initiation of a robust specific immune response against OMVs and thus against the pathogen of interest (Zhu et al. 2021).

## 4 Immune Responses to OMVs

Due to their small size from 20 to 250 nm (Qing et al. 2019; Schwechheimer and Kuehn 2015), OMVs can traffic directly into lymphatic vessels to reach the lymph nodes without the need of carrying APCs. Within lymph nodes, OMVs are presented by migrating (i.e., dendritic cells or DCs) or resident APCs (i.e., subcapsular sinus macrophages) to activate adaptive immune cells such as T lymphocytes (helper and cytotoxic) and B lymphocytes (antibody producing plasma cells), key players of the cellular and humoral immunity (Bachmann and Jennings 2010; Zhu et al. 2021). Consequently, OMVs are a great advantage as antigen carriers in vaccine development because their size allows their uptake by APCs or direct entry into lymph vessels to induce a potent adaptive response (Bachmann and Jennings 2010).

Following vaccination or infection, naive T and B cells give rise to effectors cells, short‐lived and fully activated, driving the immediate specific immune response. In addition to effector cells, parts of T and B cells will acquire a long‐lived memory phenotype, becoming the cellular support of the adaptive immune response. Each clone of these memory cells is antigen‐specific and can rapidly differentiate into effector cells and expand upon booster vaccination or re‐exposure to a pathogen expressing the same antigen. This enables the host to protect itself more effectively and rapidly than an unvaccinated host.

**FIGURE 2:**
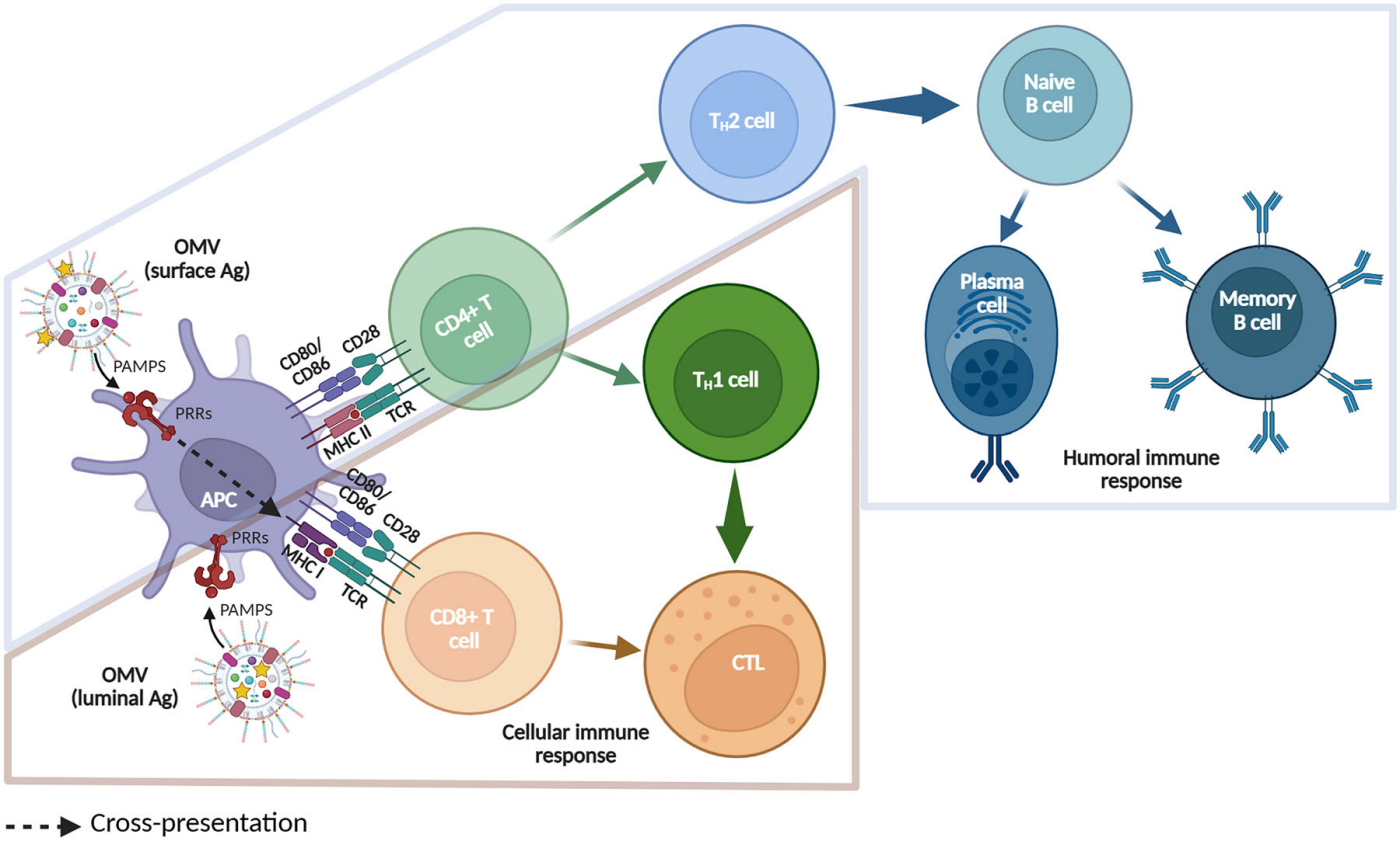
Adaptive immune response induced by OMVs. The PAMPs of OMVs are recognized by pattern recognition receptors (PRRs) such as TLRs and NOD‐like receptors (NLRs) of antigen‐presenting cells (APCs). In the case of luminal antigens, APCs internalize these antigens and present them to CD8+ T cells through their major histocompatibility complex I (MHC I). The recognition of the antigen and the expression of co‐stimulatory molecules CD80/CD86 and CD28 elicit the activation of CD8+ T cells into cytotoxic T lymphocytes (CTLs), leading to the induction of the cellular immune response. In the case of surface antigens, APCs internalize these antigens to present them through their MHC II to CD4+ T cells. These cells are activated by the co‐stimulation between CD80/CD86 and CD28 leading to their polarization into Th2 cells. Th2 cells activate naive B cells which differentiate into plasma cells and memory B cells, orchestrators of the humoral immune response. Moreover, APCs have the ability to present surface antigen through their MHC I to CD8+ T cells via the process of cross‐presentation (illustrated with the black dotted arrow), inducing a cellular immune response. Created with Biorender.

Both humoral and cellular immune responses have to be considered during vaccine development. Indeed, it is considered that the cellular response should be triggered to target an intracellular pathogen, while the humoral response should be elicited to fight against extracellular pathogens. Both arms of the adaptative immune response are interconnected and most of the time required for an optimal immune response. OMVs have the advantage of being able to induce both responses. Indeed, an antigen presented on the surface of OMVs will be accessible to B cells to trigger mainly the humoral response directed against extracellular pathogens (Gerritzen et al. 2017; Leitner et al. 2013; van der Ley et al. 2021; Zhang, Yang, et al. 2018). In contrast, an antigen exported to the lumen of OMVs will induce mainly the cellular response directed against intracellular pathogens (Fantappiè et al. 2014; Gerritzen et al. 2017). However, some studies showed that surface antigens induced the cellular response (Huang et al. 2016; Rappazzo et al. 2016; Schetters et al. 2019), while luminal antigens induced the humoral response (Muralinath et al. 2011). In the latter case, it was suggested that phagocytosis disrupted OMVs integrity, releasing the antigen outside of OMVs and allowing its recognition by B cells, further triggering the humoral response (Fantappiè et al. 2014). This might be also due to the mechanism of cross‐presentation, where DCs internalize and process surface antigens and present them *via* their major histocompatibility complex I (MHC I) to cytotoxic CD8+ T lymphocytes, instead of the usual MHC II pathway to CD4+ T or B cells. This process of cross‐presentation is essential for the activation of the cellular immune response (Embgenbroich and Burgdorf 2018; Zhu et al. 2021) (Figure 2).

The immune response necessary for vaccine effectiveness depends on the pathogen. It is therefore important to adapt the vaccination strategy to the most appropriate global immune orientation: Th1 (the cellular response, with IgG2a>IgG1 type antibodies) or Th2 (the humoral response, with IgG2a<IgG1 antibodies). In this context, the possibility of influencing the location of the antigen on OMVs is a key parameter. However, it should be noted that the orientation of the immunity toward Th1 or Th2 response is never totally exclusive. Several studies have shown that both humoral and cellular responses were elicited following vaccination with OMVs containing luminal (König et al. 2021; Li, Wang, Sun, Cimino, et al. 2021) or surface antigens (Bhaumik et al. 2023; Raeven et al. 2020).

Another factor to consider for an appropriate induction of the immune response is the route of administration. Indeed, depending on the route of administration, the same vaccine may induce different immune responses. For example, intraperitoneal, intramuscular or subcutaneous OMVs administrations are known to elicit systemic humoral and/or cellular responses, with the production of IgG antibodies in the serum of the host (Kim et al. 2013; van der Ley et al. 2021; Weyant et al. 2023). However, the systemic response is not the most efficient therapeutic way to target mucosa‐infecting pathogens. In this case, the humoral immune response mediated by secretory IgA antibodies (sIgA) at mucosal membranes should be preferred (Holmgren and Czerkinsky 2005). To this aim, only the mucosal route is known to effectively induce such sIgA antibodies. For example, it has been shown that the mucosal response can be reached by intranasal or oral vaccination with OMVs or bioengineered bacterial strains to protect against respiratory or intestinal infections (Iannino et al. 2022; van der Ley et al. 2021).

## 5 The Different Types of OMVs Used as Vaccines

The type of OMVs used as a vaccine should depend on the targeted pathogen. For example, OMVs from a wild‐type (WT) pathogenic bacterium will induce an immune response specifically targeting surface‐exposed PAMPs (O‐antigen, OMPs, etc.). In addition, it is possible to modify genetically the OMVs‐producing bacteria to obtain an OMVs‐based vaccine platform harbouring various homologous or heterologous antigens, triggering immune responses against a broader range of pathogens. This section will give examples of OMVs from WT pathogenic bacteria and bioengineered strains used for experimental or commercialized vaccines.

### 5.1 Examples of OMVs From WT Pathogenic Bacteria

As they are non‐replicative and consequently safer than the deactivated whole pathogens, OMVs‐based vaccines have already been used for human or animal immunization, thus presenting an interest for a ‘One Health’ approach. It is crucial to note that OMVs from WT pathogenic bacteria (WT‐OMVs) display an LPS with an O‐antigen that is specific to the serogroup of their parent bacteria. Such OMVs produced directly from WT bacterial pathogens have been used as vaccines, as summarized in Table 1. One example is an OMVs vaccine against avian pathogenic *E. coli* (APEC). APEC strains colonize the respiratory system of poultry, triggering severe respiratory infections and systemic fatal diseases commonly called avian colibacillosis. Avian colibacillosis causes epidemics in poultry husbandries, generating huge economic losses in the poultry industry worldwide (Guabiraba and Schouler 2015). The main treatment for colibacillosis is the use of antibiotics, which contributes to the emergence of multidrug‐resistant *E. coli*. The most prevalent serogroups associated with colibacillosis outbreaks are O1, O2 and O78 (Wang et al. 2019). 10‐day‐old chickens were vaccinated twice intramuscularly, 2 weeks apart, with 75 µg of OMVs purified from an APEC strain belonging to the serogroup O78. This immunization resulted in a titre of IgG specifically recognizing LPS and OMPs of APEC O78 together with a bactericidal activity against APEC O78 in the serum of the vaccinated chickens. In addition, vaccinated chickens were protected against colibacillosis induced by a challenge with the corresponding APEC O78 strain (Wang et al. 2019) (Table 1). Similarly, 7‐day‐old chicks were immunized with two intramuscular injections, 1 week apart, with 50 µg of OMVs purified from an APEC strain belonging to the serogroup O2. This vaccine has been associated with both humoral and cellular immune responses in the vaccinated chickens and was protective against the APEC O2 strain in challenge experiments (Hu, Liu, et al. 2020) (Table 1). However, both studies targeted O78 or O2 antigens, whereas various virulent APEC serogroups are highly prevalent in husbandries, necessitating vaccines directed against several O‐antigens. To this aim, chickens were immunized with an equal mix of 50 µg of total OMVs produced from APEC strains belonging to serogroups O1, O2 and O78. This multivalent vaccine was administered three times at 1‐week intervals via the intramuscular route in chickens from 7‐day‐old. The vaccinated chickens developed both humoral and cellular immune responses protecting them against all targeted APEC serogroups O1, O2 and O78 (Hu, Li, et al. 2020) (Table 1).

**TABLE 1:** Overview of OMVs utilization from preclinical trials to commercialized vaccines.

Targeted pathogen	OMVs‐producing bacteria	Type of OMVs	Antigen of interest	Antigen location, display system^a^	Model	Relevant results	Reference
*Acinetobacter baumannii*	*Escherichia coli* DH5α	OMVs from bioengineered bacteria (b‐OMVs)	Omp22	Surface, ClyA	Mouse 2 × 50 µg, s.c.	Induction of a cellular response Reduction of the bacterial load in the lungs, spleen, liver, and kidneys Protection against *A. baumannii* lethal challenge	(Huang et al. 2016)
Avian pathogenic *E. coli* (APEC) O78	APEC O78	OMVs from WT pathogenic bacteria (WT‐OMVs)	LPS O78	Surface	Chick 2 × 75 µg, i.m.	Production of anti‐LPS and anti‐OMPs IgGs Bactericidal activity of the serum Protection against APEC O78	(Wang et al. 2019)
APEC O2	APEC O2	WT‐OMVs	LPS O2	Surface	Chick 2 × 50 µg, i.m.	Induction of humoral and cellular responses Bactericidal activity of the serum Protection against APEC O2	(Hu, Liu, et al. 2020)
APEC O1, O2 and O78	APEC O1, APEC O2 and APEC O78	Mix of WT‐OMVs	LPS O1, LPS O2, LPS O78	Surface	Chick 3 × 50 µg, i.m.	Induction of humoral and cellular responses Cross‐protection against multi‐serogroup APEC O1, O2 and O78	(Hu, Li, et al. 2020)
*Bordetella parapertussis*	*B. parapertussis*	WT‐OMVs Or WT‐OMVs mixed to antigens	Pertactin, OmpQ, porins or Tetanus and diphtheria toxoids	Surface	Mouse 2 × 3 µg, i.p.	Protection against a challenge with *B. parapertussis* AR7269 or *B. pertussis* 18323 strains	(Bottero et al. 2013)
*B. pertussis*	*B. pertussis*	WT‐OMVs mixed to antigens	Tetanus and diphtheria toxoids and Spike	Surface	Mouse 2 × 3 µg, i.m. or i.n.	Induction of a cellular immune response (and a mucosal response from intranasal inoculations)	(Pschunder et al. 2024)
*B. pertussis*	*B. pertussis*	WT‐OMVs	LPS, BrkA, Fim2/3, FHA, Prn, Ptx, Vag8	Surface	Mouse 2 × 4 µg, i.n.	Induction of humoral, mucosal and cellular responses Prevention of *B. pertussis* colonization in the lungs, trachea and the nasal cavity	(Raeven et al. 2020)
Enterohemorrhagic *E. coli* (EHEC) O157:H7	*E. coli* K12 ∆*tolR::cat*	b‐OMVs	MC001, homologous to the lipid A deacylase (LpxR)	Outer membrane, signal sequence of MC001	Mouse 1 × 10 µg and 2 × 5 µg, i.p.	Induction of an anti‐MC0001 IgG response Reduction of the EHEC O157:H7 load in feces, colon and caecum tissues	(Rojas‐Lopez et al. 2019)
EHEC O26:H11	*E. coli* BL21 Δ*ompF*	b‐OMVs	Int280	Lumen, signal sequence of PelB and surface with Lpp‐OmpA protein	Mouse 3 × 1 µg, i.p.	Induction of systemic IgG response Earlier intestinal clearance of *Citrobacter rodentium*	(Garling et al. 2025)
*Helicobacter pylori*	*Salmonella enterica* serovar Typhimurium Δ*rfbP* Δ*fliC* Δ*fljB* Δ*ompA*	b‐OMVs	UreB and CagA	Surface, Hbp signal sequence	Mouse 2 × 100 µg, *p.o*.	Induction of humoral and cellular responses Protection against intestinal damages and *H. pylori* infection	(Liu et al. 2024)
*Neisseria meningitidis* serogroup B	*N. meningitidis* NZ98/254	b‐OMVs	PorA, NHBA, fHbp, NadA	Surface, signal sequence of each antigen	Human	Commercialized vaccine Lower incidence than before the commercialization of the vaccine, with a decrease of 51% of the cases number caused by *N. meningitidis* serogroup B in 2021 in Europe	(*Bexsero* *|* European Medicines Agency 2024; European Centre for Disease Prevention and Control (ECDC) 2023)
*Pseudomonas aeruginosa*	*P. aeruginosa* PAO1	WT‐OMVs	OMPs and LPS	Surface	Mouse 3 × 30 µg + AlPO_4_ adjuvant, i.m.	Induction of a predominantly humoral response and a cellular response Reduction of *P. aeruginosa* colonization, inflammatory state and tissue damage in the lung Protection against *P. aeruginosa* PAO1 strain and XN‐01, BJ‐15, KM‐9 clinical strains	(Zhang, Yang, et al. 2018)
*P. aeruginosa*	*P. aeruginosa*‐m14	b‐OMVs	PcrV‐HitA_T_	Lumen, β‐lactamase signal sequence	Mouse 2 × 50 µg, i.m.	Induction of humoral and cellular responses Protection against PA103 strain Cross‐protection against *P. aeruginosa* PAO1 serotype O5 strain and the AMC‐PA10 clinical isolate	(Li, Wang, Sun, Cimino, et al. 2021)
*Shigella*	*S. flexneri* 2a, *S. flexneri* 3a, *S. flexneri* 6, *S. sonnei* phase I	Mix of WT‐OMVs	OMPs and LPS	Surface	Mouse 3 × 20 µg, *p.o*.	Induction of humoral, mucosal and cellular responses Protection against inflammatory intestinal symptoms Protection against *S. dysenteriae* type 1 A1, *S. flexneri* 2a NK3809, *S. flexneri* 3a NK3758, *S. flexneri* 6 NK4025, *S. boydii* type 2 NK4023 and *S. sonnei* phase I NK3918 strains	(Bhaumik et al. 2023)
*Staphylococcus aureus*	*E. coli* BL21(DE3)Δ*60*	b‐OMVs	ClfA_Y338A_‐LukE and SpA_KKAA_‐Hla_H35L_	Lumen, Lpp signal sequence	Mouse 3 × 20 µg + 2 mg/mL Alum, i.p.	Induction of humoral and cellular responses Protection from *S. aureus* challenge in the skin, sepsis and kidney abscess models	(König et al. 2021)
Group A *Streptococcus pyogenes* (GAS)	*E. coli* BL21(DE3) *ΔompA*	b‐OMVs	Slo and SpyCEP	Lumen, OmpA signal sequence	Mouse 3 × 25 µg, i.p.	Induction of a predominantly cellular response Inhibition of Slo‐mediated hemolysis and SpyCEP hydrolytic activity on IL‐8 Protection against GAS	(Fantappiè et al. 2014)
*Streptococcus pneumoniae*	*Salmonella enterica* Serovar Typhimurium strain χ9281	b‐OMVs	PspA	Lumen, β‐lactamase signal sequence	Mouse 4 × 50 µg, i.n.	Induction of humoral and mucosal responses Protection against *S. pneumoniae*	(Muralinath et al. 2011)
*Vibrio cholerae*	*V. cholerae* O1	WT‐OMVs	OMPs and LPS	Surface	Mouse 3 × 25 µg, i.n.	Induction of humoral response Reduction of the colonization of *V. cholerae* O1, no cross‐protection against serogroup O139	(Leitner et al. 2013)
H1N1 Influenza A	*E. coli* Nissle 1917 Δ*nlpI*	b‐OMVs	M2e	Surface, ClyA	Mouse 2 × 40 µg, s.c.	Induction of a cellular response Protection against H1N1 influenza A virus	(Rappazzo et al. 2016)
SARS‐CoV‐2	*N. meningitidis* HI5	b‐OMVs	Spike	Surface, mCramp	Hamster 2 × 15 µg, i.n. or i.m.	Safe vaccination Induction of an anti‐Spike IgG response Neutralization of the virus No lung lesion after SARS‐CoV‐2 challenge Authorization to enter clinical phase I trial	(van der Ley et al. 2021; Avacc_10_COVID‐19_N. meningitidis OMVs.pdf 2025)

Abbreviations: i.m., intramuscular administration; i.n., intranasal administration; i.p., intraperitoneal administration; *p.o*., *per os* administration; s.c., subcutaneous administration.

^a^ The display system is described if applicable.

Another example of OMVs from WT pathogenic bacteria that have been tested for vaccination is a mix of OMVs against the major causative agents of bacillary dysentery in humans, *Shigella flexneri* and *Shigella sonnei* (Bhaumik et al. 2023). This OMVs vaccine has been tested in mice. Three oral administrations at 2‐week intervals of an equal mix of 20 µg of total OMVs produced from *S. flexneri* serotypes 2a, 3a and 6 and *S. sonnei* serotype phase I elicited humoral, mucosal and cellular responses. Vaccinated mice were protected against a challenge with *S. dysenteriae* type 1 A1*, S. flexneri* 2a NK3809, *S. flexneri* 3a NK3758, *S. flexneri* 6 NK4025, *S. boydii* type 2 NK4023 and *S. sonnei* phase I NK3918 strains (Bhaumik et al. 2023) (Table 1).

Whooping cough is a respiratory infectious disease caused by *Bordetella pertussis*. Although individuals of all ages can become infected, young children are particularly at risk. Two types of commercial vaccines have been developed: one based on whole, non‐replicating cells of the pathogen (the ‘wP’ vaccine) and the other on purified *B. pertussis* antigens (the acellular pertussis, or ‘aP’ vaccine). However, due to the significant adverse effects associated with wP vaccines and the limited ability of aP vaccines to reduce bacterial colonization of the upper respiratory tract, a new generation of vaccines based on OMVs has been proposed (Rudi et al. 2024). The Dutch company IntraVacc demonstrated the efficacy and immunogenicity of a vaccine candidate (Avacc 3) based on OMVs from *B. pertussis* in a mouse model. Mice vaccinated intranasally or subcutaneously developed high systemic IgG antibody levels and a systemic Th1/Th2‐related cytokine response. Moreover, intranasal immunization also induced the production of mucosal IgA antibodies. Finally, vaccinated mice were protected against *B. pertussis* challenge compared to unvaccinated control mice (Avacc_3_Pertussis_Outer Membrane Vesicle vaccine.pdf 2025). This vaccine was recently licensed to Beijing Zhifei Lvzhu Biopharmaceutical Company for distribution in Africa, South America, China and other Asian countries.

*Vibrio cholerae* is also a major human pathogen responsible for severe and epidemic diarrhoea around the world. All age groups are affected, but young children have a higher mortality rate than other groups. O1 and O139 are the main *V. cholerae* serogroups associated with epidemics. However, there is no vaccine that provides long‐term immunity against them (Leitner et al. 2013). Nevertheless, experimental vaccines based on OMVs produced from *V. cholerae* have been developed against cholera and tested in mice (Leitner et al. 2013; Sinha et al. 2015). Mice receiving three intranasal doses at 2‐week intervals of 25 µg of OMVs from *V. cholerae* O1 developed a humoral response against LPS O1. It has been observed that the passage of antibodies from breast milk to neonatal mice protected them against a challenge with *V. cholerae* O1. However, after a challenge with a strain of *V. cholerae* from the serogroup O139, neonatal mice were colonized similarly to the non‐immunized group. These results suggested that the immunization with OMVs from a strain of *V. cholerae* serogroup O1 induced antibodies principally directed against LPS O1 and did not protect against a strain of *V. cholerae* serogroup O139 (Leitner et al. 2013) (Table 1).

### 5.2 Examples of OMVs Produced by Bioengineered Bacteria

As described above, OMVs from WT Gram‐negative bacteria decorated with LPS trigger an immune response mainly directed against the O‐antigen. As we discussed for APEC and *V. cholerae*, targeting the O‐antigen for vaccination could be too narrow to induce an immune response against a given bacterial pathogen with a broad range of serogroups. In addition, a response targeting only the O‐antigen may not lead to sufficient protection, as the O‐antigen may not be the first antigen recognized by the host. Consequently, to improve the response against a targeted pathogen, homologous or heterologous antigens can be added to OMVs by bioengineering the producing bacteria. Examples of OMVs vaccines produced by bioengineered bacteria (b‐OMVs) are detailed below and summarized in Table 1.

*Neisseria meningitidis* is a human pathogen responsible for meningitis and septicemia, which can lead to death. Serogroup B was the main serogroup isolated in 68% of cases in Europe in 2012 (European Centre for Disease Prevention and Control (ECDC) 2023). The first intention treatment is the use of antibiotics, consequently associated with the emergence of multidrug‐resistant bacteria. To fight against this serogroup, preventive vaccination has been broadly developed, including the 4CMenB OMVs‐based vaccine (Bexsero) (Taha et al. 2023). *N. meningitidis* NZ98/254 was genetically engineered to expose several virulence factors on the surface of OMVs, including the Neisserial Heparin Binding Antigen (NHBA), the factor H binding protein (fHbp) and the Neisseria adhesin A (NadA), together with the naturally surface‐exposed OMP PorA (Serruto et al. 2012) (Table 1). This vaccine has been approved for human use in Europe in 2013 (*Bexsero* *|* European Medicines Agency 2024), and in the United States in 2015 (MacNeil et al. 2015). Other countries such as Australia, Canada, Chile, Brazil and Argentina subsequently approved the vaccine (Villena et al. 2018). Since its approval and according to the epidemiologic report of invasive meningococcal disease, the 4CMenB vaccine seems to have a positive effect in view of the decrease to 51% of the number of cases caused by serogroup B in 2021 in Europe (European Centre for Disease Prevention and Control (ECDC) 2023).

Other experimental OMVs vaccines were developed to fight against *P. aeruginosa* (Li, Wang, Sun, Cimino, et al. 2021; Li, Wang, Sun, Guan, et al. 2021; Zhang, Yang, et al. 2018). *P. aeruginosa* is an opportunistic bacterium which is responsible for pneumonia, surgical infection and bacteremia particularly in immunocompromised patients with cancer, AIDS or cystic fibrosis (Li, Wang, Sun, Cimino, et al. 2021). The *P. aeruginosa* PA103 strain has been mutated for several virulence factors to reduce the toxicity of its OMVs. The resulting strain, PA‐m14, was then bioengineered to express the fusion protein Bla‐PcrV‐HitA_T_ which consisted of the signal sequence of the β‐lactamase (Bla) fused to the structural protein of the type III secretion system (T3SS) PcrV and to the ferric iron‐binding periplasmic protein HitA. The expression of this fusion allowed the export of the bivalent antigen PcrV‐HitA_T_ to the periplasm of PA‐m14, resulting in the production of OMVs carrying PcrV‐HitA_T_ in their lumen (OMVs‐PH). Two intramuscular immunizations of mice with 50 µg of OMVs‐PH at 3‐week intervals induced both humoral and cellular immune responses. Vaccinated mice were protected against a *P. aeruginosa* PA103 challenge. Interestingly, OMVs‐PH provided broader protection against the *P. aeruginosa* PAO1 serotype O5 strain and the AMC‐PA10 clinical isolate (Li, Wang, Sun, Cimino, et al. 2021) (Table 1).

Bioengineered OMVs were also used as vaccines to target Gram‐positive bacteria, such as *Staphylococcus aureus* responsible for community‐ or hospital‐acquired infections (Irene et al. 2019; König et al. 2021; Sun et al. 2023). In one study, the *E. coli* BL21(DE3)Δ*60* strain was used to produce two chimeric proteins, ClfA_Y338A_‐LukE and SpA_KKAA_‐Hla_H35L,_ fused to the Lpp signal sequence, which allowed their translocation to the lumen of OMVs (CLSH‐OMVs_Δ_ *_60_*). Mice immunized three times, 2 weeks apart, with 20 µg of CLSH‐OMVs_Δ_ *_60_* developed both humoral and cellular responses directed against all four antigens. Immunization elicited functional antibodies, as judged by their activity in opsonophagocytosis, inhibition of Hla‐mediated haemolysis, inhibition of LukED‐mediated leukocyte killing and inhibition of ClfA‐mediated *S. aureus* binding to fibrinogen. Finally, mice were protected against *S. aureus* challenge in a skin, sepsis and kidney abscess models (König et al. 2021) (Table 1).

OMVs‐based vaccines can display viral antigens such as the Spike protein to target SARS‐CoV‐2. This virus is responsible for hypoxia, respiratory failure, or multiorgan dysfunction in the most severe cases (Avacc_10_COVID‐19_N. meningitidis OMVs.pdf, 2025). *N. meningitidis* HI5 strain was bioengineered to display the mCramp‐Spike fusion protein on the surface of OMVs. Mice were immunized twice, 3 weeks apart, with 15 µg of OMVs‐mCramp‐Spike. Following intranasal or intramuscular administrations, mice developed an anti‐Spike IgG response. Mice immunized intranasally produced also an anti‐Spike IgA response. Further experiments on hamsters showed that two intranasal or intramuscular immunizations, three weeks apart, with 15 µg of OMVs‐mCramp‐Spike elicited the production of anti‐Spike IgGs, and protected the immunized hamsters against a challenge with the SARS‐CoV‐2 (van der Ley et al. 2021). The company IntraVacc demonstrated the safety, tolerability, and immunogenicity of this vaccine in rabbits. Based on these promising results, IntraVacc obtained the authorization to enter clinical phase I trials with this OMVs‐based vaccine (Avacc_10_COVID‐19_N. meningitidis OMVs.pdf, 2025) (Table 1).

To produce OMVs containing homologous or heterologous antigens, various bioengineering approaches have been developed, which are described in the next section.

## 6 Strategies for Distinct Display and Location of Antigens in OMVs

The modification of OMVs properties through the bioengineering of the bacterial producer has caught the interest of several research teams in the world. Indeed, several bioengineering strategies have been developed to export antigens of interest either to the lumen or to the surface of OMVs to drive their immunogenicity.

### 6.1 Export of Antigens to the Surface of OMVs

**FIGURE 3:**
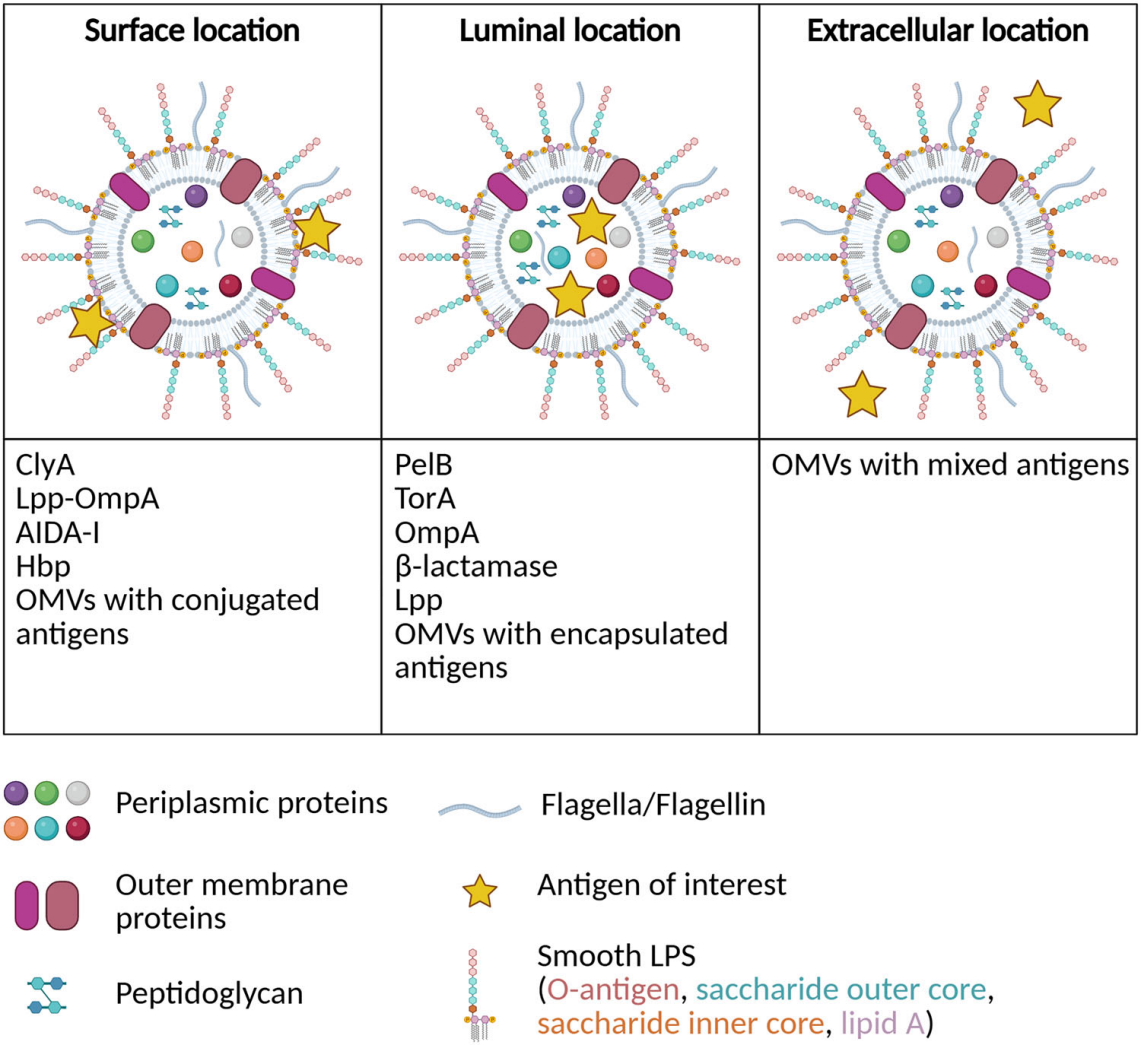
Strategies to associate antigens with OMVs from WT bacteria. Antigens can be displayed on the surface of OMVs through fusion with ClyA, Lpp‐OmpA, the adhesin involved in diffuse adherence‐I (AIDA‐I), Hbp, or by conjugation to OMVs (left panel). Antigens can be translocated in the lumen of OMVs through fusion with signal peptides such as those of PelB, TorA, OmpA, β‐lactamase, Lpp or by encapsulation into OMVs (middle panel). Finally, antigens can simply be mixed with OMVs (right panel). Created with Biorender.

For exogenous or endogenous antigens that are not naturally exported to the surface of the bacterial OM, mechanisms have been developed to display them on the surface of the OM and thus of OMVs. They usually involve a fusion between the antigen of interest and a homologous protein (or a portion of protein) naturally exported to the OM of the bacteria. ClyA, Lpp‐OmpA, AIDA‐I, and Hbp derived fusion systems are the most described in the literature and commonly used to export antigens to the surface of OMVs (Figure 3).

#### 6.1.1 Cytolysin A

Cytolysin A (or ClyA, also known as HlyE or SheA) is a 34 kDa protein found in *Salmonella* Typhi, *S*. Paratyphi A, and pathogenic or non‐pathogenic *E. coli* (Murase 2022). ClyA is a toxin that is first exported in its soluble monomeric form from the cytoplasm to the periplasm and then incorporated into the OM prior to its export into OMVs as an oligomer (Murase 2022). This oligomer, whose activity depends on the redox status, forms a pore in the host cell membrane causing haemolysis (Murase 2022; Wai et al. 2003). The capacity of ClyA to be exported to OMVs led to the development of a molecular tool using translational fusions of ClyA with antigens of interest to be displayed on the surface of OMVs. It is noteworthy that these fusions abolish the oligomerization of ClyA, thus preventing its toxic activity. Interestingly, translational fusions of ClyA with proteins such as GFP, β‐lactamase, organophosphorus hydrolase (OPH) and β‐galactosidase have a high capacity to be exported to the surface of OMVs without altering the function of the protein of interest, particularly when it is fused to the C‐terminal part of ClyA (Kim et al. 2008). Since this discovery, OMVs‐based vaccines containing engineered ClyA‐antigens have been increasingly used. For example, the fusion of ClyA with the M2e protein from Influenza A virus resulted in the exposure of M2e on the surface of OMVs. Mice vaccinated with such OMVs were successfully protected against H1N1 Influenza A virus in challenge experiments (Rappazzo et al. 2016). Another study demonstrated that the immunization of mice with OMVs carrying ClyA fused to the Omp22 protein from *A. baumannii* efficiently protected mice from a lethal challenge with this pathogen (Huang et al. 2016).

#### 6.1.2 The Lpp‐OmpA Combination

Another system developed for the presentation of an antigen on the surface of OMVs is a fusion protein combining Lpp and OmpA. Lpp is a lipoprotein that is first translocated from the cytoplasm to the periplasm via the secretory (Sec) system. Lpp is then matured by fatty acylation and taken in charge by the LolABCDE system which transports and anchors Lpp to the OM (Tokuda and Matsuyama 2004). Lpp is sufficient to export a protein to the OM. Indeed, a Lpp‐Bla fusion containing the Lpp signal sequence, the first nine amino acids (AA) of mature Lpp and the Bla enzyme was efficiently exported to the OM of *E. coli* JA221 strain. However, Bla was localized on the periplasmic side, and not on the outer leaflet of the OM as required for a presentation of the antigen on the surface of OMVs (Ghrayeb and Inouye 1984).

To correct this, OmpA was added to the Lpp portion. OmpA is an outer membrane protein found in the *Enterobacteriaceae* family and is composed of eight transmembrane domains with its C‐terminus facing the periplasm (Freudl et al. 1990). To optimize the export of β‐lactamase to the surface of the OM, a truncated OmpA including its transmembrane strands 3 to 7 (amino acids 46 to 159) was fused at its C‐terminal part to Bla (Francisco et al. 1992). However, in the absence of the eighth transmembrane strand of OmpA containing the export signal to the OM (Klose et al. 1988; Xian et al. 2007), the OmpA(46‐159)‐Bla fusion could not be exported to the OM. To avoid this, the authors fused the Lpp signal sequence and the first nine AA of mature Lpp at the N‐terminus of the recombinant protein OmpA(46‐159)‐Bla. The resulting chimeric protein Lpp‐OmpA(46‐159)‐Bla was successfully taken up by the LolABCDE system and exported to the OM. With this fusion, Bla was exposed on the bacterial surface of the *E. coli* strain JM109 (Francisco et al. 1992).

This system has been widely tested to display other proteins such as the single‐chain Fv antibody fragment (Francisco, Campbell, et al. 1993), the Cex cellulase and the Cex cellulose‐binding domain (Francisco, Stathopoulos, et al. 1993). It is noteworthy that the Lpp‐OmpA(46‐66) construct containing a shorter OmpA portion also successfully exported Bla to the surface of *E. coli* (Georgiou et al. 1996).

To develop an intimin‐enriched OMVs vaccine aimed at controlling the intestinal carriage of EHEC in ruminants, we fused the Lpp‐OmpA(46‐159) hybrid protein to the C‐terminal domain of intimin (Int280), enabling its export to the surface of OMVs. A vaccine combining OMVs displaying surface‐exposed Int280 and OMVs containing luminal Int280 (via the PelB peptide signal; see below) was tested as a proof of concept in mice using *Citrobacter rodentium*, which shares a similar intimin‐based adhesion mechanism with EHEC. Three intraperitoneal immunizations performed at 2‐week intervals with OMVs‐Int280 elicited a strong anti‐intimin IgG response in mice (Garling et al. 2025). Notably, vaccinated mice exhibited a shorter duration of *C. rodentium* fecal shedding compared to unvaccinated controls (Garling et al. 2025). This OMVs‐Int280 vaccine therefore represents a promising candidate for reducing EHEC intestinal carriage and faecal shedding in ruminants.

Another experimental OMVs‐based vaccine employed the Lpp‐OmpA hybrid protein to display the receptor binding domain (RBD) of the SARS‐CoV‐2 Spike protein on the surface of OMVs (LOR‐OMVs). Three intranasal administrations of LOR‐OMVs in mice induced a Spike‐specific humoral response capable of neutralizing a lentivirus pseudotyped with the SARS‐CoV‐2 Spike protein (Thapa et al. 2021).

#### 6.1.3 Adhesin Involved in Diffuse Adherence (AIDA‐I)

The adhesin involved in diffuse adherence‐I (AIDA‐I) from enteropathogenic *E. coli* is an autotransporter belonging to the family of type 5 secretion system (T5SS). Fusion proteins were developed containing the AIDA‐I N‐terminal signal peptide, followed by the protein of interest and an AIDA‐I translocator domain which forms a β‐barrel domain in the OM to mediate translocation and surface exposure (Nicchi et al. 2021). Interestingly, this system succeeded in exposing on the surface of *E. coli*, the *E. coli* BL21(DE3) lipoproteins LolB, LptE and Pal, as well as the *N. meningitidis* NZ98/254 lipoproteins CsgG, BamE and an additional putative lipoprotein (Nicchi et al. 2021). Furthermore, the efficiency of the autotransporter domain of AIDA‐I to export proteins with a proper conformation to the bacterial surface was also demonstrated with the β‐lactamase activity (Lattemann et al. 2000). A mixed vaccine has been developed consisting of a live *S*. Typhimurium SL3261 strain expressing KMP‐11 or 08.1190 antigens from *Leishmania donovani*, combined with *E. coli* OMVs displaying both antigens on their surface via the AIDA‐I system. Mice primed with *S*. Typhimurium SL3261 strain and boosted with OMVs exhibited up to a 40‐fold increase in specific IgG against KMP‐11 or 08.1190 antigens, compared to mice receiving a single administration of the *S*. Typhimurium SL3261 strain alone (Schroeder and Aebischer 2009).

#### 6.1.4 The Autotransporter Haemoglobin Protease (Hbp)

The haemoglobin protease (Hbp) from *E. coli* belongs to the T5SS family. This protein was engineered into an efficient secretion system to export proteins to the surface of bacteria (Jong et al. 2014). As for the AIDA‐I display system, the antigen of interest is fused between the N‐terminal part containing the signal sequence of Hbp and the C‐terminal part corresponding to a β‐barrel domain which anchors in the bacterial OM (Jong et al. 2014). This system successfully exported the pneumococcal surface protein A (PspA) (Kuipers et al. 2015), the ovalbumin (OVA) (Schetters et al. 2019) or the monoavidin protein (Weyant et al. 2023) to the surface of OMVs used for vaccination purposes. Interestingly, in bioengineered *S*. Typhimurium, Hbp had the potential to export simultaneously multiple heterologous antigens from either *Mycobacterium tuberculosis* (Daleke‐Schermerhorn et al. 2014) or *Helicobacter pylori* (Liu et al. 2024) to the surface of OMVs. When administered to mice, such OMVs induced potent immune responses that conferred protection against these two targeted pathogens (Daleke‐Schermerhorn et al. 2014; Liu et al. 2024).

#### 6.1.5 Comparison of Distinct Systems to Export Antigens to the Surface of OMVs

The efficiency of the AIDA‐I system in exporting several lipoproteins was compared to that of the Lpp‐OmpA fusion protein. It was observed that the AIDA‐I system was better to display *N. meningitidis* lipoproteins on the surface of *E. coli*, without promoting cell lysis and with less variation in the export of lipoproteins compared to the Lpp‐OmpA(46‐159) fusion protein (Nicchi et al. 2021).

The quantity of antigens exported to the surface of OMVs is an important parameter for vaccine development. This quantity varies depending on the surface display system used. For example, the AIDA‐I protein was more efficient than the Lpp‐OmpA(46‐159) fusion in exporting heterologous lipoproteins to the surface of *E. coli* while it was the reverse for the export of homologous lipoproteins (Nicchi et al. 2021). However, high expression of fusion proteins can be challenging, as it may be toxic for the producing bacteria, as observed with the Lpp‐OmpA(46‐159) fused to *E. coli* lipoproteins or with the monoavidin protein (Nicchi et al. 2021; Weyant et al. 2023). It should be noted that, although representing only 1% of total proteins, the fusion protein ClyA‐Omp22 was able to induce a protective immune response in mice (Huang et al. 2016).

An advantage of Lpp‐OmpA(46‐159) and AIDA‐I is that they are able to export both small and large proteins (Georgiou et al. 1996; Nicchi et al. 2021). However, dimerization of proteins can have an impact on their export. For example, Lpp‐OmpA(46‐159) could not export the dimerized alkaline phosphatase PhoA (Stathopoulos et al. 1996). In this case, the AIDA‐I system could be useful, as is capable of exporting a dimerized protein, that is, the sorbitol dehydrogenase (Jose and von Schwichow 2004).

Another difficulty in exposing antigens on the surface of bacteria is to keep them in a native conformation for the induction of an appropriate immune response. Indeed, humoral‐associated immune cells recognize and induce an antibody response against the 3D‐structure of the antigen. The correct folding of the exposed antigen is therefore crucial for the efficient neutralization of the targeted pathogen. It was observed that the Bla protein exported via Lpp‐OmpA(46‐159) retained up to 30% of its enzymatic activity (Francisco et al. 1992) and a similar enzymatic activity was obtained for surface‐displayed Bla using the AIDA‐I system (Lattemann et al. 2000).

In conclusion, it is important to consider the variety of surface display systems because the efficiency of export can be influenced by the nature of the antigen, its size and folding. Besides, the expression level is important to ensure sufficient antigen on the OMVs surface to induce an immune response without inducing toxicity for the producing bacteria. To counteract this toxicity or to increase the yield of the antigen of interest, antigens can be mixed with OMVs (Gerritzen et al. 2017) (Figure 3). In this case, OMVs are used for their intrinsic adjuvant properties to stimulate immune cells, while the soluble antigens serve to trigger a specific immune response. For example, Slo and SpyCEP antigens of Group A *Streptococcus* were mixed with *E. coli* OMVs. This mixture elicited an IgG response in mice similar to that obtained from either SpyCEP‐OMVs or OMVs‐Slo produced from engineered *E. coli* strains (Fantappiè et al. 2014). Another example is a vaccine composed of *B. pertussis* OMVs mixed with three heterologous antigens: the tetanus toxoid, diphtheria toxoid and the SARS‐CoV‐2 Spike protein (Pschunder et al. 2024). Two intranasal or intramuscular administrations of this formulation to mice induced a strong IgG response against all heterologous antigens, with a Th1‐skewed profile. Intranasal administration also elicited a mucosal IgA response. This robust immune response highlights the adjuvant capacity of OMVs compared to purified antigens and underscores their effectiveness in generating a comprehensive immune response (Pschunder et al. 2024) (Table 1).

*B. parapertussis* is another causative agent of whooping cough. However, its incidence is likely underestimated due to non‐mandatory surveillance and clinical symptoms that are difficult to distinguish from those of *B. pertussis*. Current commercial vaccines are ineffective at preventing lung colonization by *B. parapertussis* (Bottero et al. 2013). To address this, a new experimental vaccine, TdapOMVsBpp, was developed using *B. parapertussis* OMVs mixed with tetanus and diphtheria toxoids. Mice received two intraperitoneal injections of 3 µg of TdapOMVsBpp, 1 week apart. Remarkably, vaccinated mice were protected not only against intranasal challenge with *B. parapertussis* strain AR729 but also against *B. pertussis* strain 18323. Together, these results indicate that TdapOMVsBpp is a promising vaccine candidate capable of protecting humans against whooping cough caused by *B. parapertussis* and *B. pertussis* (Bottero et al. 2013) (Table 1).

Another strategy to bypass the expression of an antigen by OMVs‐producing bacteria is to mix the antigen with OMVs and link them together, through chemical or spontaneous conjugation (Gerritzen et al. 2017; Weyant et al. 2023) (Figure 3). For example, the mCramp‐Spike fusion protein was spontaneously conjugated to OMVs due to the affinity between the antimicrobial peptide mCramp and the LPS of OMVs. Hamsters immunized with OMVs‐mCramp‐Spike produced anti‐Spike IgGs, which protected them against a challenge with SARS‐CoV‐2 (van der Ley et al. 2021). Another example is the use of an avidin‐based vaccine antigen crosslinking (AvidVax) system. The surface of OMVs was remodeled with a synthetic antigen‐binding protein (SNAP), including ClyA, Lpp‐OmpA, Hbp or AIDA‐I, fused to monoavidin, a biotin‐binding protein which can link biotinylated antigens of interest. Therefore, SNAP‐OMVs can be decorated with a wide range of biotinylated antigens, such as peptides, proteins, carbohydrates, glycolipids, glycoproteins, haptens, lipids and nucleic acids. Mice immunized with SNAP‐OMVs decorated with biotinylated OMP from *Chlamydia muridarum* (Sx‐Cm‐MOMP) produced anti‐Cm‐MOMP IgGs which neutralized *C. muridarum* infectivity. The AvidVax technology is therefore a highly modular and versatile platform for application as vaccines (Weyant et al. 2023).

### 6.2 Export of Antigens to the Lumen of OMVs

The display of an antigen of interest on the surface of OMVs induces mainly the humoral response, which is usually the most interesting for vaccinating against extracellular pathogens. However, exporting an antigen to the lumen of OMVs could be also of interest for vaccinations targeting intracellular pathogens and/or for protecting the antigen against degradation by proteases.

To export an antigen to the lumen of OMVs, it should be first exported into the periplasmic space of the bacteria. In Gram‐negative bacteria, the crossing of the IM by proteins is an active mechanism orchestrated by two major pathways: the general Sec pathway and the twin‐arginine translocation (Tat) pathway. These two systems recognize proteins via their signal sequences. The structure of both signal sequences is similar, with a positively charged N‐region, a hydrophobic H‐region and a C‐region containing the recognition site Ala‐X‐Ala for the signal peptide. In addition, the N‐region of Tat signal peptides contain a twin‐arginine motif S‐R‐R‐x‐F‐L‐K (x is a polar AA) (Frain et al. 2019). It is noteworthy that the Sec system translocates unfolded proteins whereas the Tat system translocate folded proteins across the IM (Natale et al. 2008). This implies that the Sec pathway exports most of the proteins (Frain et al. 2019).

Both systems can be considered to translocate an antigen of interest into the lumen of OMVs. To this aim, the protein of interest can be fused to various signal sequences such as those of TorA in the case of the Tat pathway, or of PelB, OmpA, Lpp and β‐lactamase (among others) for the Sec system.

#### 6.2.1 Translocation of Proteins Through the Tat System

The signal sequence of the TorA protein from the trimethylamine N‐oxide (TMAO) reductase (Frain et al. 2019) has been used to export GFP to the periplasm of *E. coli* (Albiniak et al. 2013; Thomas et al. 2001) or into the lumen of OMVs (Kesty and Kuehn 2004; Torres‐Vanegas et al. 2024), serving as a proof of concept for targeting proteins to the interior of OMVs (Figure 3). However, to our knowledge, the TorA signal sequence has not yet been exploited to export target antigens into the OMVs lumen for the development of a vaccine platform.

#### 6.2.2 Translocation of Proteins Through the Sec System

The signal sequences of various secretory proteins recognized by the Sec system have been exploited to export antigens of interest into the lumen of OMVs.

The signal sequence of the pectate lyase PelB described in *Erwinia caratovora* (Yoon et al. 2010) has been used to translocate heterologous proteins to the periplasm of *E. coli*, such as the Phospholipase A(2) from *Streptomyces violaceoruber* (Takemori et al. 2012) or the β‐lactamase (Mirzadeh et al. 2020). As described above, we recently used the PelB signal sequence to export Int280 to the lumen of *E. coli* OMVs, which were then incorporated into a mixed OMVs vaccine containing both luminal Int280 and surface‐exposed Int280 OMVs. This vaccine successfully induced an immune response in mice and promoted earlier intestinal clearance of the murine pathogen *C. rodentium* (Garling et al. 2025) (Figure 3).

The OmpA signal sequence has also been employed to export various antigens into the OMVs lumen, including the Slo and SpyCEP antigens. Mice vaccinated intraperitoneally with either Slo‐OMVs or SpyCEP‐OMVs were protected against Group A *Streptococcus* (Fantappiè et al. 2014). Similarly, the SseB antigen from *S*. Typhimurium and the H5 viral protein from Influenza A virus were delivered into OMVs using the OmpA signal sequence. Intranasal inoculation of mice with either SseB‐OMVs or H5F‐OMVs elicited both systemic and mucosal responses against SseB and Influenza A virus, respectively. Furthermore, mice vaccinated with H5F‐OMVs showed reduced Influenza A virus PR8 titre in the lungs compared to mice that received either empty OMVs or PBS (Carvalho et al. 2019) (Figure 3).

The Lpp signal sequence has been used to deliver four *S. aureus* virulence factors into the lumen of OMVs, that is, Clumping factor A (ClfA_Y338A_), Leukocidin E (LukE), *S. aureus* protein A (SpA) and α‐hemolysin (Hla_H35L_). Specifically, two chimeric proteins, ClfA_Y338A_‐LukE and SpA_KKAA_‐Hla_H35L_, were fused to the Lpp signal sequence and expressed in *E. coli* BL21(DE3)Δ*60* strain to produce OMVs (referred to as CLSH‐OMVs_Δ_ *_60_*). Mice vaccinated intraperitoneally, three times at 2‐week intervals, with CLSH‐OMVs developed both humoral and cellular immune responses, and were protected in models of *S. aureus* skin infection, sepsis and kidney abscess (König et al. 2021) as described in the Section 5.2 ‘Examples of OMVs produced by bioengineered bacteria’ (Figure 3).

Another targeting system utilized the β‐lactamase signal sequence to export the pneumococcal surface protein A (PspA) in the lumen of OMVs for vaccine development against *Streptococcus pneumoniae* (Muralinath et al. 2011) (Figure 3). Mice intranasally immunized four times with 50 µg of OMVs‐PspA generated both humoral and mucosal responses, conferring protection in a *S. pneumoniae* sepsis model. A similar approach was employed to develop a vaccine against *P. aeruginosa* using a fusion protein composed of the type III secretion system structural protein PcrV and the ferric iron‐binding periplasmic protein HitA (Li, Wang, Sun, Cimino, et al. 2021). OMVs containing luminal PcrV‐HitAT were shown to protect mice from *P. aeruginosa* infection (Li, Wang, Sun, Cimino, et al. 2021) as also discussed in the section 5.2 ‘Examples of OMVs produced by bioengineered bacteria’.

#### 6.2.3 Comparison of Distinct Systems to Export Antigens to the Lumen of OMVs

The choice of the best system for protein translocation depends on the protein properties. First, it is important to consider whether the protein contains post‐translational modifications. Proteins requiring disulfide bonds must be in an oxidative compartment, such as the periplasm, to be properly conformed. In the cytoplasm, these proteins are in a non‐conformed state and are translocated to the oxidative periplasm ideally by the Sec pathway, before their maturation by periplasmic chaperone proteins. By contrast, proteins which do not require an oxidative environment for their maturation are usually folded in the cytoplasm, and are translocated through the IM via the Tat pathway (Frain et al. 2019). For example, the alkaline phosphatase (AP) is translocated from the reductive cytoplasm to the oxidative periplasm where it is maturated with the formation of two disulfide bonds. This translocation is mediated by the Sec system but not by the Tat transporter due to the lack of folding of this protein in the cytoplasm (DeLisa et al. 2003). However, a mutation of the *E. coli* DR473 strain making its cytoplasm an oxidative compartment allowed the correct folding of the AP in the cytoplasm and its efficient translocation by the Tat transporter (DeLisa et al. 2003).

Another study compared the efficacy of the signal sequences of PelB, OmpA, DsbA, FhuD, MdoD and YcdO to translocate the lipase lipBJ10 of the *Pseudomonas fluorescens* BJ‐10 strain into the periplasm of *E. coli* (Zhang, Lu, et al. 2018). Although all the resulting fusion proteins were exported into the periplasm of *E. coli*, the DsbA signal peptide was optimal to export a functional and soluble LipBJ10 into the periplasm. Finally, there is no universal signal peptide for a specific recombinant protein that ensures its successful translocation. Consequently, the identification of an ideal signal peptide for efficient export must be studied on a case‐by‐case basis for each new recombinant protein (Zhang, Lu, et al. 2018).

To bypass issues related to the expression and folding of fusion proteins in bacteria, the loading of heterologous antigens of interest in the lumen of OMVs can be achieved through encapsulation (Figure 3). Different methods have been described for EVs, including active loading (using detergent or electroporation) or passive loading. These encapsulation methods are more or less effective, depending on the size and biochemical properties of the molecules to be encapsulated (Gerritzen et al. 2017). Encapsulation of the *Brevundimonas diminuta* phosphotriesterase (PTE) within *E. coli* OMVs has been described. This protein was fused to SpyCatcher, itself covalently attached to SpyTag which was fused to the periplasmic facing part of OmpA. Compared to free PTE, the activity of encapsulated PTE was preserved in various storage conditions tested, such as time of storage (up to 14 days), at elevated temperature, upon several freeze‐thaw cycles or after lyophilization (Alves et al. 2016).

Strategies involving encapsulation, mixing or conjugation of antigens in the lumen or on the surface of OMVs offer a better control of the concentration of these antigens in OMVs compared to approaches relying on the expression of fusion proteins from OMVs‐producing bacteria. However, the main advantage of expressing antigens directly in bacteria is that it enables their export either to the surface or into the lumen of OMVs. Indeed, unlike conjugation, mixing or encapsulation methods, this strategy does not require antigen purification—a significant benefit, as antigen purification is time‐consuming and costly at the vaccine development scale.

Moreover, it is entirely feasible to use multiple antigen display systems within a single OMV (Wo et al. 2023), or in a mixture of OMVs (Garling et al. 2025) to combine both luminal and surface‐exposed antigens in the same vaccine formulation. For example, the receptor binding domain (RBD) of SARS‐CoV‐2 was fused to the C‐terminal region of OmpA for export into the lumen of OMVs (RBD‐OMVs). Simultaneously, the fusion protein ClyA‐NG06, where NG06 corresponds to the core peptide of the RBD, was displayed on the surface of the same OMVs. This strategy resulted in the creation of the bivalent vaccine NR‐OMVs, which simultaneously carried luminal RBD and surface‐exposed NG06. Mice vaccinated intraperitoneally three times with NR‐OMVs exhibited significantly higher anti‐RBD IgG titres compared to mice vaccinated with OMVs containing either NG06 or RBD alone (Wo et al. 2023).

Finally, OMVs are a versatile tool for vaccine development. Numerous systems have been developed that easily export homologous or heterologous proteins either inside or to the surface of OMVs to target specific pathogens and trigger distinct immune responses. However, it is noteworthy that the efficiency of each display system depends on the antigen of interest, its expression and conformation in bacteria used for OMVs production. Different systems should therefore be tested for each antigen to choose the best one that should be used for vaccine production.

## 7 Challenges Associated With Increased and Large‐Scale Production, Purification, Safety and Long‐Term Stability of OMVs

The increased and large‐scale production of OMVs for vaccine development remains challenging due to the complexity of scaling up the production process while preserving OMVs integrity, purity, safety and stability. In this section, we will first examine the main obstacles to increased and large‐scale production of OMVs‐based vaccines, along with potential strategies to overcome them. We will then address the challenges related to OMVs purification and long‐term storage, as well as associated safety concerns.

### 7.1 Mechanisms of OMVs Overproduction and Bioengineering of Bacterial Strains to Enhance the Production of OMVs Vaccines

Large production of OMVs from bacterial cultures can be challenging because a low yield of OMVs is usually produced in standard culture conditions of Gram‐negative bacteria (Balhuizen et al. 2021; Zhu et al. 2021). It is well‐known that environmental signals such as iron concentration, contact with antibiotics, growth temperature or pH are crucial factors to consider for OMVs production (Bauwens, Kunsmann, Karch, et al. 2017; Bauwens, Kunsmann, Marejková, et al. 2017; Katsui et al. 1982; Prados‐Rosales et al. 2014). Besides, a long cultivation time should be excluded as it favours the production of other types of EVs, such as explosive vesicles, which may contain DNA potentially transferable to recipient bacteria (Klimentová and Stulík 2015). Growth conditions should thus be optimized to favour OMVs production. The use of bioengineered bacterial strains is another efficient way to improve the synthesis and release of OMVs. In this section, we will first describe the parameters that are known to influence the amount and composition of OMVs such as oxygen and iron levels, antibiotic exposure, culture medium, incubation time and the use of bioreactors for bacterial cultivation. Then, we will describe two mechanisms leading to OMVs overproduction, related to the asymmetry of the OM and its link to the peptidoglycan, which can be exploited for the bioengineering of bacterial strains optimized for OMVs production.

#### 7.1.1 Culture Conditions and Versatility of OMVs Synthesis

Although OMVs are spontaneously produced by Gram‐negative bacteria, several studies demonstrated that environmental factors influence the amount of OMVs produced, their size, their composition or even their properties to induce inflammation in the host. One of the main factors affecting OMVs production is the oxygen tension of the bacterial culture. For example, 4‐fold more OMVs were produced by *N. meningitidis* when cultured with an air saturation of 150% compared to 30% (Gerritzen et al. 2018).

In line with oxygen, iron concentration is a factor usually pleiotropic during bacterial growth. As a consequence, an increase of OMVs production was observed in iron‐depleted media. For example, OMVs production by *M. tuberculosis* increased 2‐fold in iron‐restricted medium compared to iron‐rich medium (Prados‐Rosales et al. 2014). The increase of OMVs production under iron starvation was also observed with *H. pylori* (Keenan and Allardyce 2000), enterohemorrhagic *E. coli* (EHEC) O157:H7 and EHEC O104:H4 (Bauwens, Kunsmann, Marejková, et al. 2017). These OMVs will also be enriched with siderophore receptors since upon iron limitation, such as host inflammation, the bacterial OM is usually enriched with siderophore receptors (Prados‐Rosales et al. 2014).

The addition of antibiotics in the culture medium can also favour OMVs production. For example, supplementation of the quinolone ciprofloxacin in EHEC O157:H7 and EHEC O104:H4 cultures resulted in the increase of OMVs production by 250‐ and 183‐fold, respectively, compared to cultures without ciprofloxacin (Bauwens, Kunsmann, Karch, et al. 2017). The increase of OMVs by ciprofloxacin was proposed to result from the activation of the SOS response (Yang et al. 2023). Furthermore, by eliciting the SOS response, ciprofloxacin will induce the lytic cycle of prophages which may enrich OMVs with toxins. Indeed, in EHEC O157:H7 and O104:H4 strains, ciprofloxacin activated the expression of the Shiga toxin 2a (Stx2a)‐encoding gene harbored by the Stx prophage, resulting in OMVs enriched with Stx2a (Bauwens, Kunsmann, Karch, et al. 2017).

A certain attention needs to be paid to the conditions under which OMVs are produced, as this has an impact on the nature of the vesicles produced. An accumulation of EVs was observed during the stationary state compared to the exponential phase (Baeza et al. 2021; Tashiro et al. 2010). At the stationary phase, the decrease of nutrient concentration affects protein expression and increases the concentration of misfolded protein in the periplasm, thus favouring EVs production with modifications in their content (Baeza et al. 2021). It is also important to note that the stationary phase promotes bacterial lysis and release of cytoplasmic compounds leading to the formation of vesicles other than OMVs, such as O‐IMVs or EOMVs (Klimentová and Stulík 2015). Finally, an extensive study on *H. pylori* demonstrated that the duration of growth before OMVs purification modified the amount of OMVs produced, their size, but even more importantly their composition in protein and their ability to induce the production of IL‐8 by human gastric adenocarcinoma cells. Indeed, *H. pylori* accumulated more OMVs after 72 h than 16 h, and these OMVs were less heterogeneous in size, had a different protein composition and were more immunostimulatory (Zavan et al. 2019).

Other parameters have been described to modify OMVs production such as the culture medium (Choi et al. 2014; Santos et al. 2012), the pH (Bauwens, Kunsmann, Marejková, et al. 2017) or even the culture temperature (McMahon et al. 2012; Roden et al. 2012).

The versatility of OMVs production and composition could be an advantage for vaccine development as it is possible to simply modify the properties of the OMVs produced. However, it could also be a drawback as the culture conditions for homogeneous batches of OMVs vaccine must be tightly monitored.

#### 7.1.2 Production of OMVs in Bioreactors

The use of bioreactors for OMVs production offers several advantages, including an improved control over key parameters such as temperature, pH, and oxygen levels. Moreover, transitioning from batch to fed‐batch or continuous culture systems enhances biomass productivity, leading to higher OMVs yields and reduced production costs. For instance, the annual production of OMVs from *N. meningitidis* was shown to increase by 4.9‐fold in continuous cultures and 1.4‐fold in fed‐batch cultures, compared to traditional batch processes. Importantly, continuous OMVs production was reproducible, with no significant changes in OMVs concentration, size or protein composition (Gerritzen, Stangowez, et al. 2019).

Further yield improvements have been achieved by optimizing specific bioreactor parameters, such as oxygen levels and cysteine concentration (Gerritzen et al. 2018; Gerritzen, Salverda 2019; Waterbeemd, D.e, Zomer, van den Ijssel, et al. 2013). These studies underscore the strong potential of bioreactor‐based cultivation for the industrial‐scale production of OMVs. As an example, OMVs from *S. sonnei* produced in a 5‐liter fermenter yielded quantities estimated to be sufficient for manufacturing 400,000 doses of the MenZB vaccine (the precursor of Bexsero vaccine) in a 100‐liter fermenter setup (Scorza et al. 2012).

However, large‐scale cultivation or stress conditions applied to enhance OMVs production can lead to the accumulation of unwanted components such as DNA or cytoplasmic proteins, due to increased bacterial lysis (Gerritzen, D.e, Zomer, Kaaijk, et al. 2019; Waterbeemd, D.e, Zomer, van den Ijssel, et al. 2013; Scorza et al. 2012). Notably, DNA contamination was significantly reduced in continuous cultures compared to batch and fed‐batch systems (Waterbeemd, D.e, Zomer, van den Ijssel, et al. 2013). The key challenge thus lies in striking an optimal balance between maximizing OMVs yields to reduce production costs, while preserving vesicle purity and integrity.

#### 7.1.3 Disruption of the OM Asymmetry

**FIGURE 4:**
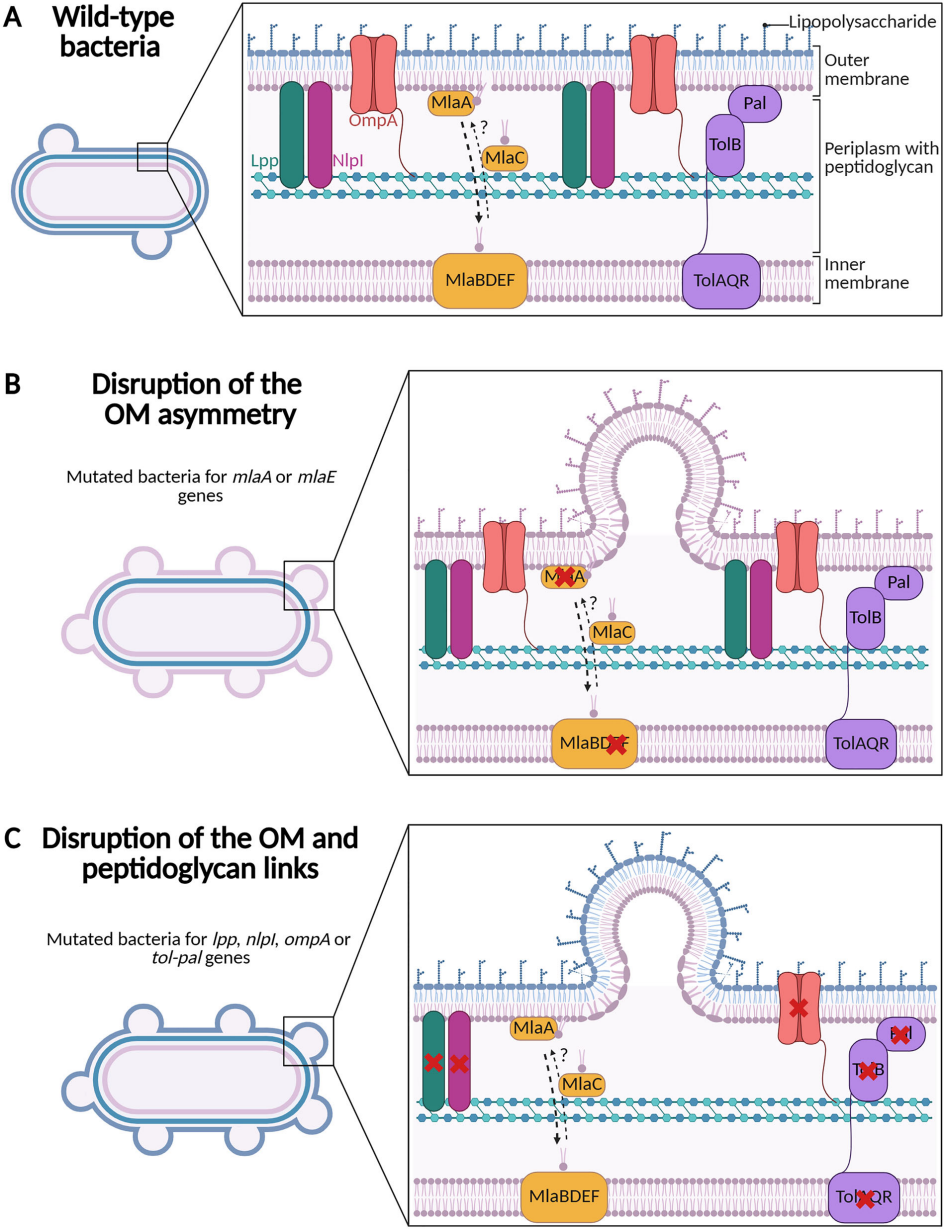
Bioengineering of *E. coli* to favour the OMVs production. (A) Wild‐type bacteria with a functional MlaABCDEF‐mediated retrograde/anterograde transport of phospholipids (PLs) from the outer membrane (OM) to the inner membrane (IM), which maintains the asymmetry of the OM lipid bilayer. Links between the OM and peptidoglycan mediated by OmpA, lipoproteins Lpp and NlpI, and the Tol‐Pal system also ensure the cell envelope integrity. (B) Mutation of *mlaA* or *mlaE* genes leads to the disruption of the OM asymmetry. PLs are not transported correctly from the OM to the IM, causing an enrichment of PLs in the OM and leading to the overproduction of OMVs. Consequently, the membrane of OMVs is also enriched with PLs. C) Mutations of *lpp*, *nlpI*, *ompA* or *tol‐pal* genes disrupt the links between the OM and peptidoglycan, resulting in a local instability of the cell envelope leading to hypervesiculation. Created with Biorender.

In addition to utilizing bioreactors and optimizing cultivation parameters, higher OMVs yields can be achieved by using bacteria deleted for genes encoding proteins involved in OM asymmetry. This promotes enhanced budding of the bacterial OM. As described above, the OM is a distinctive element of Gram‐negative bacteria. It is highly asymmetric, with inner and outer leaflets composed with PLs and mainly LPS, respectively. Due to this asymmetry and LPS properties, the OM acts as a permeation barrier against both polar and lipophilic molecules. As it is crucial for the cell envelope integrity, the asymmetry of the OM is highly regulated. In various Gram‐negative bacteria, one of the main systems described so far for tight maintenance of the OM lipid bilayer asymmetry is the maintenance of lipid asymmetry (Mla) system (Figure 4A).

The Mla system is present in different species of Gram‐negative bacteria such as *E. coli, S*. Typhimurium*, V. cholerae*, *Yersinia enterocolitica, Haemophilus influenzae, N. meningitidis* (Roier et al. 2016). This system includes the MlaBDEF complex anchored in the IM, the MlaA protein anchored in the OM and the periplasmic transporter MlaC. Whether this system transports the phospholipids from the IM to the OM or inversely remains controversial (Malinverni and Silhavy 2009; Hughes et al. 2019), although there is much more evidence that the transport might be retrograde, that is, from the OM to the IM (Malinverni and Silhavy 2009). Once phospholipids are exported to the outer leaflet of the OM, they are transferred by MlaA to MlaC, which transports them to the MlaBDEF complex for their incorporation into the IM (Malinverni and Silhavy 2009) (Figure 4A).

Loss‐of‐function mutations in the Mla system, and particularly the MlaA or MlaE proteins, modify the asymmetry of the OM and the bacterial integrity, inducing OMVs production (Ojima et al. 2020, 2021). Furthermore, as the lipid composition of OM is modified, the composition of OMVs is consequently altered (Roier et al. 2016) (Figure 4B).

#### 7.1.4 Disruption of the Links Between the OM and Peptidoglycan

The integrity of Gram‐negative bacteria cell envelope is also maintained by a peptidoglycan synthesis/degradation balance and by the presence of links between peptidoglycan and the OM. An alteration in either one or both systems induces the overproduction of OMVs. So far, the main proteins that have been described which are involved in these functions, and thus in OMVs overproduction, are the outer membrane protein OmpA, the lipoproteins Lpp and NlpI, and the Tol‐Pal system (Schwechheimer et al. 2013) (Figure 4C).

As described before, OmpA is the major OMP in *E. coli* and other *Enterobacteriaceae*. Its C‐terminal part anchors covalently peptidoglycan to the OM, allowing the bacteria to maintain their cell envelope integrity (Wang 2002) (Figure 4A). The mutation of *ompA* in *E. coli* destabilized the links between the peptidoglycan and the OM and favoured the production of OMVs (Schwechheimer et al. 2014; Sonntag et al. 1978). This phenomenon seems to be conserved among Gram‐negative bacteria as the *ompA* mutation has also been associated with a hypervesiculation phenotype in *A. baumannii, S*. Typhimurium and *V. cholerae* strains (Deatherage et al. 2009; Moon et al. 2012; Song et al. 2008).

In *E. coli*, other proteins also covalently link the peptidoglycan to the OM. The major lipoproteins Lpp and NlpI have a lipidic moiety at their N‐terminus anchored in the OM and a C‐terminal protein part anchored to the peptidoglycan (Mathelié‐Guinlet et al. 2020; Ohara et al. 1999; Tokuda and Matsuyama 2004) (Figure 4A). Besides, NlpI maintains peptidoglycan synthesis by inhibiting directly or indirectly the endopeptidases PBP4 and Spr involved in peptidoglycan degradation (Schwechheimer et al. 2015). The deletion of the *lpp* and *nlpI* genes has been correlated with increased OMVs production. For example, a deletion of the *lpp* gene led to an overproduction of 100‐fold and about 5‐fold more OMVs in *E. coli* and *Salmonella*, respectively, compared to the respective WT strains (Deatherage et al. 2009; Schwechheimer et al. 2014). Besides, a hypervesiculation by 7‐ and 100‐fold was observed with the *E. coli* K‐12 BW25113 Δ*nlpI* and MK8A44 Δ*nlpI* strains, respectively, compared to the respective WT strains (McBroom et al. 2006; Ojima et al. 2020).

Finally, the Tol‐Pal system is a complex formed between the OMP Pal, the periplasmic protein TolB anchored to the peptidoglycan and the TolA‐TolQ‐TolR complex located within the IM (Bouveret et al. 1995; Derouiche et al. 1995; Schwechheimer et al. 2013). TolB interacts with both TolA and Pal, strengthening the cell envelope integrity (Clavel et al. 1998; Hirakawa et al. 2022) (Figure 4A). In *E. coli*, a deletion of *tolR* caused a decrease in the number of links between the OM and peptidoglycan (Bernadac et al. 1998) and resulted in an OMVs overproduction 33‐fold compared to the WT strain (Pérez‐Cruz et al. 2016). *H. pylori tolB* or *pal* mutants produced 600‐ or 22‐fold more OMVs than the WT strain, respectively (Turner et al. 2015). In *Salmonella*, a deletion of *tolB* or *pal* genes favoured the overproduction of OMVs about 5‐ and 8‐fold, respectively, compared to the WT strain (Deatherage et al. 2009). In *E. coli*, deletion of *tolA* or *tolQ* genes favoured the production of OMVs compared to the WT strain (Bernadac et al. 1998). In *Salmonella*, the amount of OMVs was 14‐fold higher in a *tolA* mutant than in the WT strain (Deatherage et al. 2009).

In conclusion, knowledge on the adaptation of bacteria to their environment could also be easily exploited to improve the production of OMVs by Gram‐negative bacteria. Bioengineered Gram‐negative bacteria can be used to favour OMVs production as detailed above. However, depending on the mutation, it should be noted that the composition of the OM can be altered. Mutations of *mlaA* or *mlaE* genes lead to differences in the PLs composition of the OMVs in contrast to mutations of *lpp*, *nlpI*, *ompA*, *tol‐pal* system genes. Moreover, OMVs from *mla* mutants have less LPS on their surface and may therefore be less immunogenic and adjuvant than those from WT strains, triggering a weaker immune response against the antigen of interest. On the other hand, OMVs from *mla* mutants are probably less toxic, which can be advantageous if the host is highly sensitive to LPS. Therefore, the mutation selected for bioengineering the OMVs‐producing bacteria is an important factor to consider in OMVs vaccine development.

### 7.2 Safety and Quality Concerns

OMVs‐based vaccines are held to the same rigorous safety and quality standards as other medicinal products. Key safety considerations include ensuring the purity and defined composition of the vaccine, as well as minimizing its potential toxicity. In this section, we first describe the methods used for OMVs isolation and purification aimed at removing contaminants while preserving the vesicles’ immunogenic properties. We then discuss the challenges associated with reducing the potential toxic effects of OMVs, particularly those arising from components such as LPS. Finally, we describe the stability challenges in the development of OMVs‐based vaccines.

#### 7.2.1 Challenges in Isolation and Purification of OMVs

OMVs isolation and purification processes are necessary to remove any bacterial debris, DNA, protein aggregates or bacterial surface appendages like flagella that could contaminate the OMVs fraction for vaccine use (Hong et al. 2019; Tulkens et al. 2020). Several techniques are commonly used for OMVs purification, such as ultracentrifugation, ultrafiltration, density gradient centrifugation (DGC) or size exclusion chromatography (SEC) (Castillo‐Romero et al. 2023; Klimentová and Stulík 2015). Before the OMVs purification process, bacterial cultures are centrifuged at a low speed to pellet the bacteria and the supernatant is harvested and filter‐sterilized.

The most common and the easiest process to harvest and concentrate OMVs is the ultracentrifugation (Castillo‐Romero et al. 2023; Li et al. 2020). The culture supernatant is directly centrifuged at a high speed for a few hours to pellet OMVs. This non‐specific technique can be used to efficiently harvest various types of EVs with high yield. However, using ultracentrifugation without an initial step of supernatant concentration is highly time‐consuming and resource‐intensive, as ultracentrifugation can only process very limited volumes (Torres‐Vanegas et al. 2024; Zhao et al. 2025). Moreover, OMVs purity may be compromised due to co‐purification of bacterial cell debris, bacteriophages, large protein aggregates or surface appendages such as fimbriae and flagella. Thus, additional methods like density gradient centrifugation or size exclusion chromatography are recommended to separate OMVs from contaminants (Kulp and Kuehn 2010; Klimentová and Stulík 2015; Castillo‐Romero et al. 2023).

Ultrafiltration provides a more scalable option for concentrating and purifying OMVs. Filter‐sterilized supernatants are directly processed through ultrafiltration cassettes designed to concentrate the OMVs by draining the medium and low molecular weight molecules. Ultrafiltration could be performed with large volumes of bacterial culture in a reduced time, rendering it faster and providing a higher yield of OMVs than ultracentrifugation (Reimer et al. 2021). However, due to the retention of high molecular weight particles, this technique does not provide OMVs with high purity.

OMVs precipitation (i.e., by adding salt such as ammonium sulphate) coupled with centrifugation is a simple technique to purify OMVs from any amount of bacterial culture. In this case, precipitants are directly added to the supernatant, the latter being centrifuged for harvesting OMVs. However, this technique is not commonly used as the purity of OMVs is low and the addition of salts make them less pure than ultracentrifugation or ultrafiltration with a comparable yield (Castillo‐Romero et al. 2023; Klimentová and Stulík 2015).

To increase the purity of OMVs for vaccinal use, additional techniques have been developed. Density gradient centrifugation provides an excellent separation and high purity of OMVs based on their density (Klimentová and Stulík 2015). However, this method requires a great deal of precision from the experimenter, making it time‐consuming and highly variable from user to user. The difficulty to standardize this purification is thus not ideal for large‐scale production (Collins et al. 2021).

Finally, size exclusion chromatography separates different EVs types based on their size through a column, enabling the isolation of high‐purity OMVs. Although this purification method preserves OMVs integrity and functionality, its volume capacity is limited. This makes challenging for large‐scale production if not used in combination with ultrafiltration and/or ultracentrifugation. Additionally, the resolution may not always be optimal in distinguishing OMVs from other particles with similar sizes, potentially leading to contamination of OMVs (Castillo‐Romero et al. 2023).

In conclusion, when used on their own, none of the available OMVs purification methods are ideal for large‐scale production for vaccine applications. However, depending on the intended use and the specific characteristics of the OMVs being produced and purified, combining multiple techniques can provide both high yield and purity suitable for vaccine development.

#### 7.2.2 Challenges in LPS Detoxification

As mentioned above, LPS, and specifically lipid A, is the major adjuvant being a strong potent molecule. Consequently, it is crucial to find a balance between its adjuvant property to induce a sufficient immune response and its capacity to induce a deregulated inflammatory response that could lead to sepsis, potentially fatal. It is noteworthy that animal species do not have a similar sensitivity to lipid A. Intravenous injection with doses of 0.015 and 0.025 mg of LPS/kg cause a septic shock in humans and calves, respectively. In mice, the lethal dose 50 (LD_50_) of 10 mg of LPS/kg was determined while in chickens a dose of 50 mg of LPS/kg is not lethal (Warren et al. 2010).

As the balance between immune activation and uncontrolled inflammation is critical for the development of vaccines, methods have been elaborated to overcome the lipid A toxicity. One example is the treatment of purified OMVs with detergents or EDTA for the detoxification of LPS (van der Pol et al. 2015; Zariri et al. 2016). Another example is the mutation of genes involved in the lipid A biosynthesis pathway, allowing the production of OMVs with a less toxic lipid A (Balhuizen et al. 2021; Leitner et al. 2013; van de Waterbeemd et al. 2010; Zariri et al. 2016). Detergent or EDTA detoxified‐*N. meningitidis* OMVs and OMVs obtained from mutants in the lipid A biosynthesis pathway were compared for their capacity to induce the innate immune response using HEK‐Blue hTLR4 and hTLR2 cells (Zariri et al. 2016). Although effective in reducing the toxicity of LPS, detergent treatment led to the loss of protective antigens such as lipoproteins and reduced both TLR4 and TLR2 activation (Zariri et al. 2016). In contrast, mutations in *lpxL1* and *pagL* (both genes encoding enzymes involved in lipid A acylation) led to the production of OMVs that activated less the TLR4 than the WT bacteria. These results thus showed that the treatment of OMVs with detergent or EDTA was not suitable for vaccine applications. On the other hand, modifications in the lipid A biosynthesis pathway attenuated the toxicity of OMVs without losing their immunogenic properties (Zariri et al. 2016).

Furthermore, antigens present in OMVs‐based vaccines retain their native conformation and functionality, which is important for the production of functional antibodies against pathogens. However, the presence of LPS and major OMPs on the surface of OMVs may reduce the antigen yield in OMVs, resulting in an immune response mainly directed against the O‐antigen of the LPS or OMPs. An alternative to reduce such non‐specific responses is to produce OMVs from bacterial strains containing rough LPS and/or deficient for OMPs production. These strains include *E. coli* BL21(DE3) (Studier and Moffatt 1986), which has a truncated LPS lacking the O‐antigen (Figure 1B). The adjuvanticity properties of rough LPS, but also its toxicity, are preserved as lipid A is still present. For the use of OMVs vaccines in a host highly sensitive to lipid A, the ClearColi strain (Lucigen) containing a modified lipid A could be used. The use of a strain mutated in genes involved in the synthesis of the O‐antigen and/or endogenous OMVs‐located proteins could also be used. In line with this, an *E. coli* BL21(DE3) strain producing proteome‐minimized OMVs was constructed by mutating sixty protein‐encoding genes (Zanella et al. 2021) (Figure 1B). This *E. coli* BL21(DE3)Δ*60* strain was able to produce a large amount of OMVs which elicited specific cellular or humoral responses against the different heterologous antigens exposed. Altogether, OMVs produced from this strain have the potential to be used as a novel vaccine platform (Zanella et al. 2021).

#### 7.2.3 Challenges in Maintaining the Long‐Term Stability of OMVs

Another key regulatory requirement is to ensure that vaccines remain both their effective and safe over their designated shelf life. For OMVs‐based vaccines, storage conditions can compromise vesicle integrity, potentially leading to a loss of vaccine potency over time. This represents a significant challenge when scaling up OMVs production for clinical applications. Several studies have explored strategies to enhance OMVs stability, including the use of stabilizing agents such as cryoprotectants, as well as preservation techniques like freeze‐drying and lyophilization. For example, the stability of the phosphotriesterase PTE encapsulated in OMVs (see above) was compared to that of its purified form under various stress conditions, including prolonged storage, elevated temperatures, repeated freeze‐thaw cycles, and lyophilization (Alves et al. 2016). OMVs‐encapsulated PTE retained approximately 85% of its enzymatic activity after 14 days at −80°C or 4°C, whereas the purified enzyme retained less than 10% activity under the same conditions. After 14 days of storage at room temperature (20°C), 30°C or 37°C, OMVs‐encapsulated PTE activity declined to around 40%, which still significantly exceeded the activity of purified PTE, which dropped to between 0% and 25%. Moreover, OMVs‐encapsulated PTE preserved 90% of its activity after successive freeze‐thaw cycles, and 60% after lyophilization followed by storage at RT. Importantly, NanoSight analysis with Nanoparticle Tracking and Analysis (NTA) software showed that OMVs concentration and size remained stable after freeze‐thaw cycles or lyophilization. These results suggest that OMVs exhibit substantial stability under a variety of storage conditions, with cold storage at −80°C or 4°C being the most favourable for long‐term preservation (Alves et al. 2016).

Additional studies have confirmed that OMVs derived from *N. meningitidis* group B have maintained their structural integrity, PorA concentration and antigenicity after 1 year of storage at −70°C (frozen), 4°C (liquid), or lyophilized at 4°C (Arigita et al. 2004; Waterbeemd, D.e, Zomer, Kaaijk, et al. 2013). In contrast, storage at elevated temperatures (37°C or 56°C) led to OMVs degradation and PorA denaturation, resulting in a significant loss of immunogenicity (Arigita et al. 2004). These findings indicate that OMVs are thermosensitive but remain stable under refrigerated or frozen conditions, as well as when lyophilized and stored at low temperatures (Arigita et al. 2004; Waterbeemd, D.e, Zomer, Kaaijk, et al. 2013).

Finally, the optimal storage conditions for experimental OMVs‐based vaccines align with the recommended storage guidelines for the Bexsero vaccine, which must be kept between 2°C and 8°C, whether in liquid or lyophilized form (*Meningococcal Vaccines Storage and Handling* *|* CDC 2023).

## 8 Future Prospects of OMVs‐Based Vaccine Platforms

The global rise in antimicrobial resistance (AMR) poses a major threat, potentially resulting in up to 10 million deaths annually by 2050 (O'Neill 2016). The use of OMVs vaccines to combat AMR bacteria has garnered significant interest due to their potential to prevent infections caused by bacterial pathogens. Several experimental OMVs vaccines have demonstrated their ability to induce both humoral and cellular immune responses, providing protection in mice models against AMR bacteria (Table 1). Moreover, OMVs vaccines can be engineered to target multiple bacterial strains or species simultaneously, offering the potential for broad‐spectrum protection.

Beyond targeting pathogenic and/or AMR bacteria, OMVs vaccine platforms can also be adapted to combat viral infections. Indeed, OMVs can be readily engineered to display viral antigens, enabling the rapid development of vaccines against emerging viruses such as Ebola, Bluetongue or Influenza. Experimental OMVs‐based vaccines targeting human viruses, including SARS‐CoV‐2 (Avacc_10_COVID‐19_N. meningitidis OMVs.pdf, 2025; van der Ley et al. 2021) and Zika virus (Martins et al. 2018), have already been developed, as well as OMVs vaccines against animal viruses such as avian influenza (Rappazzo et al. 2016). The flexibility and efficiency of OMVs‐based platforms position them as promising tools for rapid vaccine development in response to novel viral threats.

Cancer is the second leading cause of death worldwide (*Cancer* *|* World Health Organization 2025). Current treatment options include surgery, radiotherapy, chemotherapy or molecular targeted therapies (*Cancer* *|* World Health Organization 2025; Lee et al. 2018). However, the emergence of drug resistance and metastatic progression underscores the need for innovative anti‐tumour strategies. Although cancer vaccines have been explored, their clinical efficacy remains limited due to poor immunogenicity and potential side effects (Wang et al. 2022). OMVs offer an attractive platform for anti‐tumoral vaccines by enabling the surface display of specific tumour‐associated antigens. For instance, the ClyA signal peptide has been used to display the ovalbumin (OVA) tumour antigen on the surface of OMVs (CO‐OMVs). Mice challenged with OVA‐expressing B16 melanoma cells and vaccinated with CO‐OMVs showed significantly reduced lung metastasis compared to control animals (Cheng et al. 2021). Additionally, the SpyCatcher/SpyTag and SnoopCatcher/SnoopTag ‘Plug‐and‐Display’ systems have been used to display one or multiple antigens (e.g., TRP2, OVA1, OVA2) on the surface of OMVs *via* a fusion with ClyA. These multivalent OMVs significantly reduced lung metastasis in B16 melanoma‐bearing mice (Cheng et al. 2021). Other tumour‐targeting OMVs have utilized the ClyA signal peptide to display the single‐chain variable fragment (scFv) of the anti‐EGFR antibody panitumumab (scFv‐OMVs) (Rezaei Adriani et al. 2023), or a MerTK (myeloid‐epithelial‐reproductive tyrosine kinase) inhibitor (mU@OMVs) (Zhuang et al. 2023) to enhance anti‐tumour immunity. Intraperitoneal administration of scFv‐OMVs inhibited breast tumour growth in mice, while peritumoral injections of mU@OMVs effectively prevented tumour progression, metastasis, and recurrence (Rezaei Adriani et al. 2023; Zhuang et al. 2023). ClearColi‐derived OMVs (CC OMVs), which are LPS‐free, have also shown improved safety upon intravenous administration in mice. These OMVs promoted immune cell infiltration into tumours and enhanced tumour suppression. When engineered to display the IL‐2 variant Neo‐2/15 cytokine on their surface using the Lpp‐OmpA fusion system (CC‐Neo2/15 OMVs), they significantly improved tumour suppression and survival in a mice colorectal tumour model compared to unmodified CC‐OMVs (Chen et al. 2025). Collectively, these findings underscore the strong potential of OMVs as an innovative platform for cancer immunotherapy.

In addition, OMVs can be combined with mRNA or siRNA to serve as next‐generation anti‐tumour vaccines. This approach combines the immunogenic and delivery properties of OMVs with the versatility of RNA‐based therapeutics, enabling antigen expression in immune or tumour cells, or gene silencing involved in tumour progression. For example, siRNA targeting Kinesin Spindle Protein (KSP) was electroporated into OMVs derived from *E. coli* Δ*msbB* K‐12 W3110. These OMVs effectively silenced KSP mRNA expression *in vitro* and triggered apoptosis in cancer cells. Systemic administration of these siRNA‐loaded OMVs also led to KSP gene silencing and significant tumour regression in mice (Gujrati et al. 2014). Similarly, OMVs displaying the RNA‐binding protein L7Ae on their surface *via* the ClyA signal peptide were used for mRNA delivery into dendritic cells through specific interaction with boxC/D‐tagged mRNAs. This strategy inhibited melanoma progression and induced long‐term immune memory, protecting mice from tumour challenge (Li et al. 2022). Such RNA‐loaded OMVs represent a promising avenue for personalized cancer vaccines and next‐generation immunotherapies.

Finally, bio‐based liposomes have emerged as innovative alternative to OMVs. These artificial vesicles, composed of lipids derived from natural sources such as microorganisms, can be engineered to mimic OMVs structure and function while offering enhanced compositional control and improved safety by removing toxic components like endotoxins. Although vaccines based on synthetic liposomes (i.e., vesicles made from synthetic lipids) already exist for human and veterinary use, their production is often expensive and time‐consuming (Tollemar et al. 2001; Mischler and Metcalfe 2002; Yaguchi et al. 2009; Zhao et al. 2011; Pichon and Midoux 2013; Lecrenier et al. 2018). In this context, bio‐based liposomes represent a promising, safer and cost‐effective alternative, combining the immunostimulatory potential of OMVs with the versatility of bio‐derived lipid technologies for future vaccine development.

## 9 Conclusion

OMVs have emerged as a promising platform in vaccinology due to their innate immunogenicity, self‐adjuvanting properties, and bioengineering potential. This enables robust activation of both innate and adaptive immune pathways. OMVs' modularity allows for the incorporation of homologous or heterologous antigens through various surface and luminal display systems, including fusion proteins. This engineering flexibility facilitates the development of multivalent and cross‐protective formulations. However, antigen misfolding, impaired translocation, and epitope masking remain significant barriers, necessitating the continued optimization of expression systems, signal peptide selection, and protein folding environments. From a translational perspective, the scalability of OMVs production remains a critical constraint. Advances in strain engineering have led to hypervesiculating mutants with altered membrane asymmetry, disrupted envelope‐peptidoglycan interactions, and modified LPS biosynthesis. These mutants result in enhanced vesicle yield and reduced endotoxicity. Although bioreactor‐based systems provide controlled culture conditions that support reproducible, Good Manufacturing Practices‐compliant production, achieving the high purity and consistent composition of OMVs remains a major challenge. Beyond their use in prophylactic vaccination against bacterial and viral pathogens, including multidrug‐resistant strains, OMVs also have potential as delivery vectors for tumour‐associated antigens. This positions them at the interface of immunotherapy and nanotechnology. Their tunable immunostimulatory profile, combined with advances in synthetic biology, establishes OMVs as a promising next‐generation platform for rational vaccine design.

## Author Contributions

**Asja Garling**: Conceptualization (equal); writing–original draft (lead); writing–review and editing (equal). **Frédéric Auvray**: Writing–original draft (supporting); writing–review and editing (equal). **Mathieu Epardaud**: Writing–review and editing (equal). **Priscilla Branchu**: Conceptualization (equal); supervision (lead); writing–original draft (supporting); writing–review and editing (equal). **Éric Oswald**: Conceptualization (equal), writing ‐ original draft (supporting), writing–review and editing (equal)

## Conflicts of Interest

The authors report no conflict of interest.

## Data Availability

Data sharing is not applicable to this article as no new data were created or analyzed in this study.

## References

[jev270150-bib-0001] Adams, P. G. , L. Lamoureux , K. L. Swingle , H. Mukundan , and G. A. Montaño . 2014. “Lipopolysaccharide‐Induced Dynamic Lipid Membrane Reorganization: Tubules, Perforations, and Stacks.” Biophysical Journal 106, no. 11: 2395–2407. 10.1016/j.bpj.2014.04.016. 24896118 PMC4052278

[jev270150-bib-0002] Albiniak, A. M. , C. F. R. O. Matos , S. D. Branston , R. B. Freedman , E. Keshavarz‐Moore , and C. Robinson . 2013. “High‐Level Secretion of a Recombinant Protein to the Culture Medium With a *Bacillus subtilis* Twin‐Arginine Translocation System in *Escherichia coli* .” FEBS Journal 280, no. 16: 3810–3821. 10.1111/febs.12376. 23745597

[jev270150-bib-0003] Altindis, E. , Y. Fu , and J. J. Mekalanos . 2014. “Proteomic Analysis of *Vibrio cholerae* Outer Membrane Vesicles.” Proceedings of the National Academy of Sciences 111, no. 15: E1548–E1556. 10.1073/pnas.1403683111. PMC3992640 24706774

[jev270150-bib-0004] Alves, N. J. , K. B. Turner , I. L. Medintz , and S. A. Walper . 2016. “Protecting Enzymatic Function Through Directed Packaging Into Bacterial Outer Membrane Vesicles.” Scientific Reports 6: 24866. 10.1038/srep24866. 27117743 PMC4846811

[jev270150-bib-0005] Arigita, C. , W. Jiskoot , J. Westdijk , et al. 2004. “Stability of Mono‐ and Trivalent Meningococcal Outer Membrane Vesicle Vaccines.” Vaccine 22, no. 5: 629–642. 10.1016/j.vaccine.2003.08.027. 14741154

[jev270150-bib-0006] Arking, D. , Y. Tong , and D. C. Stein . 2001. “Analysis of Lipooligosaccharide Biosynthesis in the Neisseriaceae.” Journal of Bacteriology 183, no. 3: 934–941. 10.1128/JB.183.3.934-941.2001. 11208792 PMC94961

[jev270150-bib-0007] Aschtgen, M.‐S. , J. B. Lynch , E. Koch , J. Schwartzman , M. McFall‐Ngai , and E. Ruby . 2016. “Rotation of Vibrio Fischeri Flagella Produces Outer Membrane Vesicles That Induce Host Development.” Journal of Bacteriology 198, no. 16: 2156–2165. 10.1128/JB.00101-16. 27246572 PMC4966437

[jev270150-bib-0008] Avacc_10_COVID‐19_N. meningitidis OMVs.pdf . Consulté 8 janvier 2025. à l'adresse https://www.intravacc.nl/fileadmin/01‐Website/Downloads/Vaccsheet/Avacc_10_COVID‐19_1123_LR.pdf.

[jev270150-bib-0009] Avacc_3_Pertussis_Outer Membrane Vesicle vaccine.pdf . Consulté 31 mars 2025. à l'adresse https://www.intravacc.nl/fileadmin/01‐Website/Downloads/Vaccsheet/230530_Avacc_3_DL.pdf.

[jev270150-bib-0010] Bachmann, M. F. , and G. T. Jennings . 2010. “Vaccine Delivery: A Matter of Size, Geometry, Kinetics and Molecular Patterns.” Nature Reviews Immunology 10, no. 11: 787–796. 10.1038/nri2868. 20948547

[jev270150-bib-0011] Baeza, N. , L. Delgado , J. Comas , and E. Mercade . 2021. “Phage‐Mediated Explosive Cell Lysis Induces the Formation of a Different Type of O‐IMV in *Shewanella vesiculosa* M7T.” Frontiers in Microbiology 12: 713669. 10.3389/fmicb.2021.713669. 34690958 PMC8529241

[jev270150-bib-0012] Balhuizen, M. D. , E. J. A. Veldhuizen , and H. P. Haagsman . 2021. “Outer Membrane Vesicle Induction and Isolation for Vaccine Development.” Frontiers in Microbiology 12: 629090. 10.3389/fmicb.2021.629090. 33613498 PMC7889600

[jev270150-bib-0013] Bauman, S. J. , and M. J. Kuehn . 2006. “Purification of Outer Membrane Vesicles From *Pseudomonas aeruginosa* and Their Activation of an IL‐8 Response.” Microbes and Infection 8, no. 9: 2400–2408. 10.1016/j.micinf.2006.05.001. 16807039 PMC3525494

[jev270150-bib-0014] Bauwens, A. , L. Kunsmann , H. Karch , A. Mellmann , and M. Bielaszewska . 2017a. “Antibiotic‐Mediated Modulations of Outer Membrane Vesicles in Enterohemorrhagic *Escherichia coli* O104:H4 and O157:H7.” Antimicrobial Agents and Chemotherapy 61, no. 9: e00937‐17. 10.1128/AAC.00937-17. 28607018 PMC5571282

[jev270150-bib-0015] Bauwens, A. , L. Kunsmann , M. Marejková , et al. 2017b. “Intrahost Milieu Modulates Production of Outer Membrane Vesicles, Vesicle‐Associated Shiga Toxin 2a and Cytotoxicity in *Escherichia coli* O157:H7 and O104:H4.” Environmental Microbiology Reports 9, no. 5: 626–634. 10.1111/1758-2229.12562. 28675605

[jev270150-bib-0016] Bernadac, A. , M. Gavioli , J. C. Lazzaroni , S. Raina , and R. Lloubès . 1998. “ *Escherichia coli* Tol‐pal Mutants Form Outer Membrane Vesicles.” Journal of Bacteriology 180, no. 18: 4872–4878. 10.1128/JB.180.18.4872-4878.1998. 9733690 PMC107512

[jev270150-bib-0017] Bexsero | European Medicines Agency . 2024. https://www.ema.europa.eu/en/medicines/human/EPAR/bexsero.

[jev270150-bib-0018] Bhaumik, U. , P. Halder , D. R. Howlader , et al. 2023. “A Tetravalent *Shigella* Outer Membrane Vesicles Based Candidate Vaccine Offered Cross‐Protection Against all the Serogroups of *Shigella* in Adult Mice.” Microbes and Infection 25, no. 5: 105100. 10.1016/j.micinf.2023.105100. 36696935

[jev270150-bib-0019] Bielaszewska, M. , M. Marejková , A. Bauwens , L. Kunsmann‐Prokscha , A. Mellmann , and H. Karch . 2018. “Enterohemorrhagic *Escherichia coli* O157 Outer Membrane Vesicles Induce Interleukin 8 Production in Human Intestinal Epithelial Cells by Signaling via Toll‐Like Receptors TLR4 and TLR5 and Activation of the Nuclear Factor NF‐κB.” International Journal of Medical Microbiology 308, no. 7: 882–889. 10.1016/j.ijmm.2018.06.004. 29934223

[jev270150-bib-0020] Bishop, D. G. , and E. Work . 1965. “An Extracellular Glycolipid Produced by *Escherichia coli* Grown Under Lysine‐Limiting Conditions.” Biochemical Journal 96, no. 2: 567–576. 10.1042/bj0960567. 4953781 PMC1207076

[jev270150-bib-0021] Blackburn, S. A. , M. Shepherd , and G. K. Robinson . 2021. “Reciprocal Packaging of the Main Structural Proteins of Type 1 Fimbriae and Flagella in the Outer Membrane Vesicles of “Wild Type” *Escherichia coli* Strains.” Frontiers in Microbiology 12: 557455. 10.3389/fmicb.2021.557455. 33643229 PMC7907004

[jev270150-bib-0022] Blevins, H. M. , Y. Xu , S. Biby , and S. Zhang . 2022. “The NLRP3 Inflammasome Pathway: A Review of Mechanisms and Inhibitors for the Treatment of Inflammatory Diseases.” Frontiers in Aging Neuroscience 14: 879021. 10.3389/fnagi.2022.879021. 35754962 PMC9226403

[jev270150-bib-0023] Bos, M. P. , and J. Tommassen . 2004. “Biogenesis of the Gram‐Negative Bacterial Outer Membrane.” Current Opinion in Microbiology 7, no. 6: 610–616. 10.1016/j.mib.2004.10.011. 15556033

[jev270150-bib-0024] Bottero, D. , M. E. Gaillard , A. Errea , et al. 2013. “Outer Membrane Vesicles Derived From *Bordetella parapertussis* as an Acellular Vaccine Against *Bordetella parapertussis* and *Bordetella pertussis* Infection.” Vaccine 31, no. 45: 5262–5268. 10.1016/j.vaccine.2013.08.059. 24012570

[jev270150-bib-0025] Bouveret, E. , R. Derouiche , A. Rigal , R. Lloubès , C. Lazdunski , and H. Bénédetti . 1995. “Peptidoglycan‐Associated Lipoprotein‐TolB Interaction. A Possible Key to Explaining the Formation of Contact Sites Between the Inner and Outer Membranes of *Escherichia coli* .” Journal of Biological Chemistry 270, no. 19: 11071–11077. 10.1074/jbc.270.19.11071. 7744736

[jev270150-bib-0026] Cancer | World Health Organization . 2025. https://www.who.int/health‐topics/cancer.

[jev270150-bib-0027] Carvalho, A. L. , S. Fonseca , A. Miquel‐Clopés , et al. 2019. “Bioengineering Commensal Bacteria‐Derived Outer Membrane Vesicles for Delivery of Biologics to the Gastrointestinal and Respiratory Tract.” Journal of Extracellular Vesicles 8, no. 1: 1632100. 10.1080/20013078.2019.1632100. 31275534 PMC6598475

[jev270150-bib-0028] Castillo‐Romero, K. F. , A. Santacruz , and J. González‐Valdez . 2023. “Production and Purification of Bacterial Membrane Vesicles for Biotechnology Applications: Challenges and Opportunities.” Electrophoresis 44, no. 1–2: 107–124. 10.1002/elps.202200133. 36398478

[jev270150-bib-0029] Chen, M.‐Y. , T.‐W. Cheng , Y.‐C. Pan , et al. 2025. “Endotoxin‐Free Outer Membrane Vesicles for Safe and Modular Anticancer Immunotherapy.” ACS Synthetic Biology 14, no. 1: 148–160. 10.1021/acssynbio.4c00483. 39763210 PMC11744915

[jev270150-bib-0030] Cheng, K. , R. Zhao , Y. Li , et al. 2021. “Bioengineered Bacteria‐Derived Outer Membrane Vesicles as a Versatile Antigen Display Platform for Tumor Vaccination via Plug‐and‐Display Technology.” Nature Communications 12, no. 1: 2041. 10.1038/s41467-021-22308-8. PMC8024398 33824314

[jev270150-bib-0031] Choi, C.‐W. , E. C. Park , S. H. Yun , et al. 2014. “Proteomic Characterization of the Outer Membrane Vesicle of Pseudomonas Putida KT2440.” Journal of Proteome Research 13, no. 10: 4298–4309. 10.1021/pr500411d. 25198519

[jev270150-bib-0032] Ciesielska, A. , M. Matyjek , and K. Kwiatkowska . 2021. “TLR4 and CD14 Trafficking and Its Influence on LPS‐Induced Pro‐Inflammatory Signaling.” Cellular and Molecular Life Sciences: CMLS 78, no. 4: 1233–1261. 10.1007/s00018-020-03656-y. 33057840 PMC7904555

[jev270150-bib-0033] Clavel, T. , P. Germon , A. Vianney , R. Portalier , and J. C. Lazzaroni . 1998. “TolB Protein of *Escherichia coli* K‐12 Interacts With the Outer Membrane Peptidoglycan‐Associated Proteins Pal, Lpp and OmpA.” Molecular Microbiology 29, no. 1: 359–367. 10.1046/j.1365-2958.1998.00945.x. 9701827

[jev270150-bib-0034] Collins, S. M. , J. B. Nice , E. H. Chang , and A. C. Brown . 2021. “Size Exclusion Chromatography to Analyze Bacterial Outer Membrane Vesicle Heterogeneity.” Journal of Visualized Experiments: JoVE no.169. 10.3791/62429. PMC9266994 33871453

[jev270150-bib-0035] Daleke‐Schermerhorn, M. H. , T. Felix , Z. Soprova , et al. 2014. “Decoration of Outer Membrane Vesicles With Multiple Antigens by Using an Autotransporter Approach.” Applied and Environmental Microbiology 80, no. 18: 5854–5865. 10.1128/AEM.01941-14. 25038093 PMC4178611

[jev270150-bib-0036] Deatherage, B. L. , and B. T. Cookson . 2012. “Membrane Vesicle Release in Bacteria, Eukaryotes, and Archaea: A Conserved yet Underappreciated Aspect of Microbial Life.” Infection and Immunity 80, no. 6: 1948–1957. 10.1128/IAI.06014-11. 22409932 PMC3370574

[jev270150-bib-0037] Deatherage, B. L. , J. C. Lara , T. Bergsbaken , S. L. Rassoulian Barrett , S. Lara , and B. T. Cookson . 2009. “Biogenesis of Bacterial Membrane Vesicles.” Molecular Microbiology 72, no. 6: 1395–1407. 10.1111/j.1365-2958.2009.06731.x. 19432795 PMC2745257

[jev270150-bib-0038] DeLisa, M. P. , D. Tullman , and G. Georgiou . 2003. “Folding Quality Control in the Export of Proteins by the Bacterial Twin‐Arginine Translocation Pathway.” Proceedings of the National Academy of Sciences 100, no. 10: 6115–6120. 10.1073/pnas.0937838100. PMC156335 12721369

[jev270150-bib-0039] Derouiche, R. , H. Bénédetti , J. C. Lazzaroni , C. Lazdunski , and R. Lloubès . 1995. “Protein Complex Within *Escherichia coli* Inner Membrane. TolA N‐Terminal Domain Interacts With TolQ and TolR Proteins.” Journal of Biological Chemistry 270, no. 19: 11078–11084. 10.1074/jbc.270.19.11078. 7744737

[jev270150-bib-0040] Dhurve, G. , A. K. Madikonda , M. V. Jagannadham , and D. Siddavattam . 2022. “Outer Membrane Vesicles of *Acinetobacter baumannii* DS002 Are Selectively Enriched With TonB‐Dependent Transporters and Play a Key Role in Iron Acquisition.” Microbiology Spectrum 10, no. 2: e00293‐22. 10.1128/spectrum.00293-22. 35266817 PMC9045253

[jev270150-bib-0041] Down, K. P. , H. Nguyen , A. Dorfleutner , and C. Stehlik . 2020. “An Overview of the Non‐Canonical Inflammasome.” Molecular Aspects of Medicine 76: 100924. 10.1016/j.mam.2020.100924. 33187725 PMC7808250

[jev270150-bib-0042] Elizagaray, M. L. , M. T. R. Gomes , E. S. Guimaraes , et al. 2020. “Canonical and Non‐Canonical Inflammasome Activation by Outer Membrane Vesicles Derived From *Bordetella Pertussis* .” Frontiers in Immunology 11: 1879. 10.3389/fimmu.2020.01879. 32973778 PMC7468456

[jev270150-bib-0043] Embgenbroich, M. , and S. Burgdorf . 2018. “Current Concepts of Antigen Cross‐Presentation.” Frontiers in Immunology 9: 1643. 10.3389/fimmu.2018.01643. 30061897 PMC6054923

[jev270150-bib-0044] European Centre for Disease Prevention and Control (ECDC) . 2023. *Invasive meningococcal disease—Annual Epidemiological Report for 2021*.

[jev270150-bib-0045] Fantappiè, L. , M. de Santis , E. Chiarot , et al. 2014. “Antibody‐Mediated Immunity Induced by Engineered *Escherichia coli* OMVs Carrying Heterologous Antigens in Their Lumen.” Journal of Extracellular Vesicles 3: 00. 10.3402/jev.v3.24015. PMC4131003 25147647

[jev270150-bib-0046] Fathy Mohamed, Y. , and R. C. Fernandez . 2024. “Programming *Bordetella pertussis* Lipid A to Promote Adjuvanticity.” Microbial Cell Factories 23, no. 1: 250. 10.1186/s12934-024-02518-7. 39272136 PMC11401268

[jev270150-bib-0047] Frain, K. M. , C. Robinson , and J. M. van Dijl . 2019. “Transport of Folded Proteins by the Tat System.” Protein Journal 38, no. 4: 377–388. 10.1007/s10930-019-09859-y. 31401776 PMC6708511

[jev270150-bib-0048] Francisco, J. A. , R. Campbell , B. L. Iverson , and G. Georgiou . 1993a. “Production and Fluorescence‐Activated Cell Sorting of *Escherichia coli* Expressing a Functional Antibody Fragment on the External Surface.” Proceedings of the National Academy of Sciences USA 90, no. 22: 10444–10448. 10.1073/pnas.90.22.10444. PMC47793 8248129

[jev270150-bib-0049] Francisco, J. A. , C. F. Earhart , and G. Georgiou . 1992. “Transport and Anchoring of Beta‐Lactamase to the External Surface of *Escherichia coli* .” Proceedings of the National Academy of Sciences USA 89, no. 7: 2713–2717. 10.1073/pnas.89.7.2713. PMC48732 1557377

[jev270150-bib-0050] Francisco, J. A. , C. Stathopoulos , R. A. J. Warren , D. G. Kilburn , and G. Georgiou . 1993b. “Specific Adhesion and Hydrolysis of Cellulose by Intact *Escherichia coli* Expressing Surface Anchored Cellulase or Cellulose Binding Domains.” Bio/Technology 11, no. 4: 491–495. 10.1038/nbt0493-491. 7763519

[jev270150-bib-0051] Freudl, R. , M. Klose , and U. Henning . 1990. “Export and Sorting of the *Escherichia coli* Outer Membrane Protein OmpA.” Journal of Bioenergetics and Biomembranes 22, no. 3: 441–449. 10.1007/BF00763176. 2202726

[jev270150-bib-0052] Garcia‐Vello, P. , H. L. P. Tytgat , J. Elzinga , et al. 2024. “The Lipooligosaccharide of the Gut Symbiont Akkermansia Muciniphila Exhibits a Remarkable Structure and TLR Signaling Capacity.” Nature Communications 15, no. 1: 8411. 10.1038/s41467-024-52683-x. PMC11436972 39333588

[jev270150-bib-0053] García‐Weber, D. , and C. Arrieumerlou . 2021. “ADP‐Heptose: A Bacterial PAMP Detected by the Host Sensor ALPK1.” Cellular and Molecular Life Sciences: CMLS 78, no. 1: 17–29. 10.1007/s00018-020-03577-w. 32591860 PMC11072087

[jev270150-bib-0054] Garling, A. , C. Goursat , C. Seguy , et al. 2025. “Development of Intimin‐Enriched Outer Membrane Vesicles (OMVs) as a Vaccine to Control Intestinal Carriage of Enterohemorrhagic *Escherichia coli* .” Vaccine 52: 126899. 10.1016/j.vaccine.2025.126899. 39985970

[jev270150-bib-0055] Gasperini, G. , V. Arato , M. Pizza , B. Aricò , and R. Leuzzi . 2017. “Physiopathological Roles of Spontaneously Released Outer Membrane Vesicles of Bordetella Pertussis.” Future Microbiology 12: 1247–1259. 10.2217/fmb-2017-0064. 28980823

[jev270150-bib-0056] Georgiou, G. , D. L. Stephens , C. Stathopoulos , H. L. Poetschke , J. Mendenhall , and C. F. Earhart . 1996. “Display of Beta‐Lactamase on the *Escherichia coli* Surface: Outer Membrane Phenotypes Conferred by Lpp'‐OmpA'‐Beta‐Lactamase Fusions.” Protein Engineering 9, no. 2: 239–247. 10.1093/protein/9.2.239. 9005446

[jev270150-bib-0057] Gerritzen, M. J. H. , R. H. W. Maas , J. van den Ijssel , et al. 2018. “High Dissolved Oxygen Tension Triggers Outer Membrane Vesicle Formation by *Neisseria meningitidis* .” Microbial Cell Factories 17, no. 1: 157. 10.1186/s12934-018-1007-7. 30285743 PMC6171317

[jev270150-bib-0058] Gerritzen, M. J. H. , D. E. Martens , R. H. Wijffels , L. van der Pol , and M. Stork . 2017. “Bioengineering Bacterial Outer Membrane Vesicles as Vaccine Platform.” Biotechnology Advances 35, no. 5: 565–574. 10.1016/j.biotechadv.2017.05.003. 28522212

[jev270150-bib-0059] Gerritzen, M. J. H. , M. L. M. Salverda , D. E. Martens , R. H. Wijffels , and M. Stork . 2019a. “Spontaneously Released *Neisseria meningitidis* Outer Membrane Vesicles as Vaccine Platform: Production and Purification.” Vaccine 37, no. 47: 6978–6986. 10.1016/j.vaccine.2019.01.076. 31383485

[jev270150-bib-0060] Gerritzen, M. J. H. , L. Stangowez , B. van de Waterbeemd , D. E. Martens , R. H. Wijffels , and M. Stork . 2019b. “Continuous Production of *Neisseria meningitidis* Outer Membrane Vesicles.” Applied Microbilogy and Biotechnology 103, no. 23: 9401–9410. 10.1007/s00253-019-10163-z. PMC6867985 31676919

[jev270150-bib-0061] Ghrayeb, J. , and M. Inouye . 1984. “Nine Amino Acid Residues at the NH2‐Terminal of Lipoprotein Are Sufficient for Its Modification, Processing, and Localization in the Outer Membrane of *Escherichia coli* .” Journal of Biological Chemistry 259, no. 1: 463–467. 10.1016/S0021-9258(17)43683-07. 6368539

[jev270150-bib-0062] Gibson, B. W. , W. Melaugh , N. J. Phillips , M. A. Apicella , A. A. Campagnari , and J. M. Griffiss . 1993. “Investigation of the Structural Heterogeneity of Lipooligosaccharides From Pathogenic Haemophilus and Neisseria Species and of R‐Type Lipopolysaccharides From *Salmonella typhimurium* by Electrospray Mass Spectrometry.” Journal of Bacteriology 175, no. 9: 2702–2712. 10.1128/jb.175.9.2702-2712.1993. 8386724 PMC204573

[jev270150-bib-0063] Gill, S. , R. Catchpole , and P. Forterre . 2018. “Extracellular Membrane Vesicles in the Three Domains of Life and Beyond.” FEMS Microbiology Reviews 43, no. 3: 273–303. 10.1093/femsre/fuy042. PMC6524685 30476045

[jev270150-bib-0064] Giordano, N. P. , M. B. Cian , and Z. D. Dalebroux . 2020. “Outer Membrane Lipid Secretion and the Innate Immune Response to Gram‐Negative Bacteria.” Infection and Immunity 88, no. 7: e00920‐19. 10.1128/iai.00920-19. 32253250 PMC7309610

[jev270150-bib-0065] Guabiraba, R. , and C. Schouler . 2015. “Avian Colibacillosis: Still Many Black Holes.” FEMS Microbiology Letters 362, no. 15: fnv118. 10.1093/femsle/fnv118. 26204893

[jev270150-bib-0066] Gujrati, V. , S. Kim , S.‐H. Kim , et al. 2014. “Bioengineered Bacterial Outer Membrane Vesicles as Cell‐Specific Drug‐Delivery Vehicles for Cancer Therapy.” ACS Nano 8, no. 2: 1525–1537. 10.1021/nn405724x. 24410085

[jev270150-bib-0067] Harvey, H. A. , W. E. Swords , and M. A. Apicella . 2001. “The Mimicry of Human Glycolipids and Glycosphingolipids by the Lipooligosaccharides of *Pathogenic neisseria* and Haemophilus.” Journal of Autoimmunity 16, no. 3: 257–262. 10.1006/jaut.2000.0477. 11334490

[jev270150-bib-0068] Hirakawa, H. , K. Suzue , and H. Tomita . 2022. “Roles of the Tol/Pal System in Bacterial Pathogenesis and Its Application to Antibacterial Therapy.” Vaccines 10, no. 3: Article 3. 10.3390/vaccines10030422. PMC8953051 35335056

[jev270150-bib-0069] Holmgren, J. , and C. Czerkinsky . 2005. “Mucosal Immunity and Vaccines.” Nature Medicine 11, no. 4: S45–S53. 10.1038/nm1213. 15812489

[jev270150-bib-0070] Hong, J. , P. Dauros‐Singorenko , A. Whitcombe , et al. 2019. “Analysis of the *Escherichia coli* Extracellular Vesicle Proteome Identifies Markers of Purity and Culture Conditions.” Journal of Extracellular Vesicles 8, no. 1: 1632099. 10.1080/20013078.2019.1632099. 31275533 PMC6598517

[jev270150-bib-0071] Horstman, A. L. , and M. J. Kuehn . 2000. “Enterotoxigenic *Escherichia coli* Secretes Active Heat‐Labile Enterotoxin via Outer Membrane Vesicles.” Journal of Biological Chemistry 275, no. 17: 12489–12496. 10.1074/jbc.275.17.12489. 10777535 PMC4347834

[jev270150-bib-0072] Hu, R. , J. Li , Y. Zhao , et al. 2020a. “Exploiting Bacterial Outer Membrane Vesicles as a Cross‐Protective Vaccine Candidate Against Avian Pathogenic *Escherichia coli* (APEC).” Microbial Cell Factories 19, no. 1: 119. 10.1186/s12934-020-01372-7. 32493405 PMC7268718

[jev270150-bib-0073] Hu, R. , H. Liu , M. Wang , et al. 2020b. “An OMV‐Based Nanovaccine Confers Safety and Protection Against Pathogenic Escherichia coli via both Humoral and Predominantly Th1 Immune Responses in Poultry.” Nanomaterials 10, no. 11: Article 11. 10.3390/nano10112293. PMC7699605 33233490

[jev270150-bib-0074] Huang, W. , S. Wang , Y. Yao , et al. 2016. “Employing Escherichia coli‐derived Outer Membrane Vesicles as an Antigen Delivery Platform Elicits Protective Immunity Against Acinetobacter baumannii Infection.” Scientific Reports 6, no. 1: 37242. 10.1038/srep37242. 27849050 PMC5110958

[jev270150-bib-0075] Hughes, G. W. , S. C. L. Hall , C. S. Laxton , et al. 2019. “Evidence for Phospholipid Export From the Bacterial Inner Membrane by the Mla ABC Transport System.” Nature Microbiology 4, no. 10: 1692–1705. 10.1038/s41564-019-0481-y. 31235958

[jev270150-bib-0076] Iannino, F. , P. J. Uriza , C. M. Duarte , M. V. Pepe , M. S. Roset , and G. Briones . 2022. “Development of a Salmonella‐Based Oral Vaccine to Control Intestinal Colonization of Shiga‐Toxin‐Producing *Escherichia coli* (STEC) in Animals.” Vaccine 40, no. 8: 1065–1073. 10.1016/j.vaccine.2022.01.032. 35086742

[jev270150-bib-0077] Irene, C. , L. Fantappiè , E. Caproni , et al. 2019. “Bacterial Outer Membrane Vesicles Engineered With Lipidated Antigens as a Platform for Staphylococcus aureus Vaccine.” Proceedings of the National Academy of Sciences 116, no. 43: 21780–21788. 10.1073/pnas.1905112116. PMC6815149 31591215

[jev270150-bib-0078] Jong, W. S. , M. H. Daleke‐Schermerhorn , D. Vikström , et al. 2014. “An Autotransporter Display Platform for the Development of Multivalent Recombinant Bacterial Vector Vaccines.” Microbial Cell Factories 13, no. 1: 162. 10.1186/s12934-014-0162-8. 25421093 PMC4252983

[jev270150-bib-0079] Jose, J. , and S. von Schwichow . 2004. “Autodisplay of Active Sorbitol Dehydrogenase (SDH) Yields a Whole Cell Biocatalyst for the Synthesis of Rare Sugars.” Chembiochem 5, no. 4: 491–499. 10.1002/cbic.200300774. 15185373

[jev270150-bib-0080] Kaparakis, M. , L. Turnbull , L. Carneiro , et al. 2010. “Bacterial Membrane Vesicles Deliver Peptidoglycan to NOD1 in Epithelial Cells.” Cellular Microbiology 12, no. 3: 372–385. 10.1111/j.1462-5822.2009.01404.x. 19888989

[jev270150-bib-0081] Kaparakis‐Liaskos, M. , and R. L. Ferrero . 2015. “Immune Modulation by Bacterial Outer Membrane Vesicles.” Nature Reviews Immunology 15, no. 6: 375–387. 10.1038/nri3837. 25976515

[jev270150-bib-0082] Kashyap, D. , M. Panda , B. Baral , et al. 2022. “Outer Membrane Vesicles: An Emerging Vaccine Platform.” Vaccines 10, no. 10: 1578. 10.3390/vaccines10101578. 36298443 PMC9610665

[jev270150-bib-0083] Katsui, N. , T. Tsuchido , R. Hiramatsu , S. Fujikawa , M. Takano , and I. Shibasaki . 1982. “Heat‐Induced Blebbing and Vesiculation of the Outer Membrane of *Escherichia coli* .” Journal of Bacteriology 151, no. 3: 1523–1531. 10.1128/jb.151.3.1523-1531.1982. 7050091 PMC220434

[jev270150-bib-0084] Kawasaki, T. , and T. Kawai . 2014. “Toll‐Like Receptor Signaling Pathways.” Frontiers in Immunology 5: 00. 10.3389/fimmu.2014.00461. PMC4174766 25309543

[jev270150-bib-0085] Keenan, J. I. , and R. A. Allardyce . 2000. “Iron Influences the Expression of *Helicobacter pylori* Outer Membrane Vesicle‐Associated Virulence Factors.” European Journal of Gastroenterology & Hepatology 12, no. 12: 1267–1273. 10.1097/00042737-200012120-00002. 11192314

[jev270150-bib-0086] Kesty, N. C. , and M. J. Kuehn . 2004. “Incorporation of Heterologous Outer Membrane and Periplasmic Proteins Into *Escherichia coli* Outer Membrane Vesicles*.” Journal of Biological Chemistry 279, no. 3: 2069–2076. 10.1074/jbc.M307628200. 14578354 PMC3525464

[jev270150-bib-0087] Kim, J.‐Y. , A. M. Doody , D. J. Chen , et al. 2008. “Engineered Bacterial Outer Membrane Vesicles With Enhanced Functionality.” Journal of Molecular Biology 380, no. 1: 51–66. 10.1016/j.jmb.2008.03.076. 18511069 PMC4617544

[jev270150-bib-0088] Kim, O. Y. , B. S. Hong , K.‐S. Park , et al. 2013. “Immunization With *Escherichia coli* Outer Membrane Vesicles Protects Bacteria‐Induced Lethality via Th1 and Th17 Cell Responses.” Journal of Immunology (Baltimore, Md.: 1950) 190, no. 8: 4092–4102. 10.4049/jimmunol.1200742. 23514742

[jev270150-bib-0089] Klimentová, J. , and J. Stulík . 2015. “Methods of Isolation and Purification of Outer Membrane Vesicles From Gram‐Negative Bacteria.” Microbiological Research 170: 1–9. 10.1016/j.micres.2014.09.006. 25458555

[jev270150-bib-0090] Klose, M. , H. Schwarz , S. MacIntyre , R. Freudl , M. L. Eschbach , and U. Henning . 1988. “Internal Deletions in the Gene for an *Escherichia coli* Outer Membrane Protein Define an Area Possibly Important for Recognition of the Outer Membrane by this Polypeptide.” Journal of Biological Chemistry 263, no. 26: 13291–13296. 10.1016/S0021-9258(18)37703-2. 3047120

[jev270150-bib-0091] Kolling, G. L. , and K. R. Matthews . 1999. “Export of Virulence Genes and Shiga Toxin by Membrane Vesicles of *Escherichia coli* O157:H7.” Applied and Environmental Microbiology 65, no. 5: 1843–1848. 10.1128/AEM.65.5.1843-1848.1999. 10223967 PMC91264

[jev270150-bib-0092] König, E. , A. Gagliardi , I. Riedmiller , et al. 2021. “Multi‐Antigen Outer Membrane Vesicle Engineering to Develop Polyvalent Vaccines: The *Staphylococcus aureus* Case.” Frontiers in Immunology 12: 752168. 10.3389/fimmu.2021.752168. 34819933 PMC8606680

[jev270150-bib-0093] Kuipers, K. , M. H. Daleke‐Schermerhorn , W. S. P. Jong , et al. 2015. “Salmonella Outer Membrane Vesicles Displaying High Densities of Pneumococcal Antigen at the Surface Offer Protection Against Colonization.” Vaccine 33, no. 17: 2022–2029. 10.1016/j.vaccine.2015.03.010. 25776921

[jev270150-bib-0094] Kulp, A. , and M. J. Kuehn . 2010. “Biological Functions and Biogenesis of Secreted Bacterial Outer Membrane Vesicles.” Annual Review of Microbiology 64: 163–184. 10.1146/annurev.micro.091208.073413. PMC3525469 20825345

[jev270150-bib-0095] Kumar, H. , T. Kawai , and S. Akira . 2009. “Toll‐Like Receptors and Innate Immunity.” Biochemical and Biophysical Research Communications 388, no. 4: 621–625. 10.1016/j.bbrc.2009.08.062. 19686699

[jev270150-bib-0096] Lattemann, C. T. , J. Maurer , E. Gerland , and T. F. Meyer . 2000. “Autodisplay: Functional Display of Active β‐Lactamase on the Surface of *Escherichia coli* by the AIDA‐I Autotransporter.” Journal of Bacteriology 182, no. 13: 3726–3733. 10.1128/jb.182.13.3726-3733.2000. 10850987 PMC94543

[jev270150-bib-0097] Lecrenier, N. , P. Beukelaers , R. Colindres , et al. 2018. “Development of Adjuvanted Recombinant Zoster Vaccine and Its Implications for Shingles Prevention.” Expert Review of Vaccines 17, no. 7: 619–634. 10.1080/14760584.2018.1495565. 30028651

[jev270150-bib-0098] Lee, Y. T. , Y. J. Tan , and C. E. Oon . 2018. “Molecular Targeted Therapy: Treating Cancer With Specificity.” European Journal of Pharmacology 834: 188–196. 10.1016/j.ejphar.2018.07.034. 30031797

[jev270150-bib-0099] Leitner, D. R. , S. Feichter , K. Schild‐Prüfert , G. N. Rechberger , J. Reidl , and S. Schild . 2013. “Lipopolysaccharide Modifications of a Cholera Vaccine Candidate Based on Outer Membrane Vesicles Reduce Endotoxicity and Reveal the Major Protective Antigen.” Infection and Immunity 81, no. 7: 2379–2393. 10.1128/IAI.01382-12. 23630951 PMC3697601

[jev270150-bib-0100] Li, M. , H. Zhou , C. Yang , et al. 2020. “Bacterial Outer Membrane Vesicles as a Platform for Biomedical Applications: An Update.” Journal of Controlled Release 323: 253–268. 10.1016/j.jconrel.2020.04.031. 32333919

[jev270150-bib-0101] Li, P. , X. Wang , X. Sun , J. Cimino , Z. Guan , and W. Sun . 2021a. “Recombinant Pseudomonas Bionanoparticles Induce Protection Against Pneumonic *Pseudomonas aeruginosa* Infection.” Infection and Immunity 89, no. 11: e0039621. 10.1128/iai.00396-21. 34310892 PMC8519289

[jev270150-bib-0102] Li, P. , X. Wang , X. Sun , Z. Guan , and W. Sun . 2021b. “Outer Membrane Vesicles Displaying a Heterologous PcrV‐HitA Fusion Antigen Promote Protection Against Pulmonary *Pseudomonas aeruginosa* Infection.” mSphere 6, no. 5: e0069921. 10.1128/mSphere.00699-21. 34612675 PMC8510544

[jev270150-bib-0103] Li, Y. , X. Ma , Y. Yue , et al. 2022. “Rapid Surface Display of mRNA Antigens by Bacteria‐Derived Outer Membrane Vesicles for a Personalized Tumor Vaccine.” Advanced Materials 34, no. 20: 2109984. 10.1002/adma.202109984. 35315546

[jev270150-bib-0104] Liu, Q. , Q. Liu , J. Yi , et al. 2016. “Outer Membrane Vesicles From Flagellin‐Deficient Salmonella Enterica Serovar Typhimurium Induce Cross‐Reactive Immunity and Provide Cross‐Protection Against Heterologous Salmonella Challenge.” Scientific Reports 6, no. 1: 34776. 10.1038/srep34776. 27698383 PMC5048178

[jev270150-bib-0105] Liu, Q. , Y. Shang , L. Shen , et al. 2024. “Outer Membrane Vesicles From Genetically Engineered Salmonella Enterica Serovar Typhimurium Presenting *Helicobacter pylori* Antigens UreB and CagA Induce Protection Against *Helicobacter pylori* Infection in Mice.” Virulence 15, no. 1: 2367783. 10.1080/21505594.2024.2367783. 38937901 PMC11216100

[jev270150-bib-0106] MacNeil, J. R. , L. Rubin , T. Folaranmi , I. R. Ortega‐Sanchez , M. Patel , and S. W. Martin . 2015. “Use of Serogroup B Meningococcal Vaccines in Adolescents and Young Adults: Recommendations of the Advisory Committee on Immunization Practices, 2015.” MMWR. Morbidity and Mortality Weekly Report 64, no. 41: 1171–1176. 10.15585/mmwr.mm6441a3. 26492381

[jev270150-bib-0107] Malinverni, J. C. , and T. J. Silhavy . 2009. “An ABC Transport System That Maintains Lipid Asymmetry in the Gram‐Negative Outer Membrane.” Proceedings of the National Academy of Sciences 106, no. 19: 8009–8014. 10.1073/pnas.0903229106. PMC2683108 19383799

[jev270150-bib-0108] Manabe, T. , M. Kato , T. Ueno , and K. Kawasaki . 2013. “Flagella Proteins Contribute to the Production of Outer Membrane Vesicles From *Escherichia coli* W3110.” Biochemical and Biophysical Research Communications 441, no. 1: 151–156. 10.1016/j.bbrc.2013.10.022. 24134841

[jev270150-bib-0109] Mancini, F. , O. Rossi , F. Necchi , and F. Micoli . 2020. “OMV Vaccines and the Role of TLR Agonists in Immune Response.” International Journal of Molecular Sciences 21, no. 12: 4416. 10.3390/ijms21124416. 32575921 PMC7352230

[jev270150-bib-0110] Mandrell, R. E. , and M. A. Apicella . 1993. “Lipo‐Oligosaccharides (LOS) of Mucosal Pathogens: Molecular Mimicry and Host‐Modification of LOS.” Immunobiology 187, no. 3: 382–402. 10.1016/S0171-2985(11)80352-9. 8330904

[jev270150-bib-0111] Mandrell, R. E. , A. J. Lesse , J. V. Sugai , et al. 1990. “In Vitro and In Vivo Modification of Neisseria Gonorrhoeae Lipooligosaccharide Epitope Structure by Sialylation.” Journal of Experimental Medicine 171, no. 5: 1649–1664. 10.1084/jem.171.5.1649. 1692081 PMC2187906

[jev270150-bib-0112] Manning, A. J. , and M. J. Kuehn . 2011. “Contribution of Bacterial Outer Membrane Vesicles to Innate Bacterial Defense.” BMC Microbiology 11, no. 1: 258. 10.1186/1471-2180-11-258. 22133164 PMC3248377

[jev270150-bib-0113] Martins, P. , D. Machado , T. H. Theizen , et al. 2018. “Outer Membrane Vesicles From Neisseria Meningitidis (Proteossome) Used for Nanostructured Zika Virus Vaccine Production.” Scientific Reports 8, no. 1: 8290. 10.1038/s41598-018-26508-z. 29844457 PMC5974080

[jev270150-bib-0114] Mathelié‐Guinlet, M. , A. T. Asmar , J.‐F. Collet , and Y. F. Dufrêne . 2020. “Lipoprotein Lpp Regulates the Mechanical Properties of the *E. coli* Cell Envelope.” Nature Communications 11, no. 1: 1789. 10.1038/s41467-020-15489-1. PMC7156740 32286264

[jev270150-bib-0115] McBroom, A. J. , A. P. Johnson , S. Vemulapalli , and M. J. Kuehn . 2006. “Outer Membrane Vesicle Production by *Escherichia coli* Is Independent of Membrane Instability.” Journal of Bacteriology 188, no. 15: 5385–5392. 10.1128/JB.00498-06. 16855227 PMC1540050

[jev270150-bib-0116] McBroom, A. J. , and M. J. Kuehn . 2007. “Release of Outer Membrane Vesicles by Gram‐Negative Bacteria Is a Novel Envelope Stress Response.” Molecular Microbiology 63, no. 2: 545–558. 10.1111/j.1365-2958.2006.05522.x. 17163978 PMC1868505

[jev270150-bib-0117] McMahon, K. J. , M. E. Castelli , E. García Vescovi , and M. F. Feldman . 2012. “Biogenesis of Outer Membrane Vesicles in *Serratia marcescens* Is Thermoregulated and Can be Induced by Activation of the Rcs Phosphorelay System.” Journal of Bacteriology 194, no. 12: 3241–3249. 10.1128/JB.00016-12. 22493021 PMC3370869

[jev270150-bib-0118] Meningococcal Vaccines Storage and Handling | CDC . 2023. https://www.cdc.gov/vaccines/vpd/mening/hcp/storage‐handling.html.

[jev270150-bib-0119] Micoli, F. , and C. A. MacLennan . 2020. “Outer Membrane Vesicle Vaccines.” Seminars in Immunology 50: 101433. 10.1016/j.smim.2020.101433. 33309166

[jev270150-bib-0120] Mirzadeh, K. , P. J. Shilling , R. Elfageih , et al. 2020. “Increased Production of Periplasmic Proteins in *Escherichia coli* by Directed Evolution of the Translation Initiation Region.” Microbial Cell Factories 19: 85. 10.1186/s12934-020-01339-8. 32264894 PMC7137448

[jev270150-bib-0121] Mischler, R. , and I. C. Metcalfe . 2002. “Inflexal®V a Trivalent Virosome Subunit Influenza Vaccine: Production.” Vaccine 20: B17–B23. 10.1016/S0264-410X(02)00512-1. 12477413

[jev270150-bib-0122] Moon, D. C. , C. H. Choi , J. H. Lee , et al. 2012. “ *Acinetobacter baumannii* Outer Membrane Protein A Modulates the Biogenesis of Outer Membrane Vesicles.” Journal of Microbiology (Seoul, Korea) 50, no. 1: 155–160. 10.1007/s12275-012-1589-4. 22367951

[jev270150-bib-0123] Muralinath, M. , M. J. Kuehn , K. L. Roland , and R. Curtiss . 2011. “Immunization With Salmonella Enterica Serovar Typhimurium‐Derived Outer Membrane Vesicles Delivering the Pneumococcal Protein PspA Confers Protection Against Challenge With Streptococcus pneumoniae.” Infection and Immunity 79, no. 2: 887–894. 10.1128/IAI.00950-10. 21115718 PMC3028854

[jev270150-bib-0124] Murase, K. 2022. “Cytolysin A (ClyA): A Bacterial Virulence Factor With Potential Applications in Nanopore Technology, Vaccine Development, and Tumor Therapy.” Toxins 14, no. 2: 78. 10.3390/toxins14020078. 35202106 PMC8880466

[jev270150-bib-0125] Murase, K. , P. Martin , G. Porcheron , et al. 2016. “HlyF Produced by Extraintestinal Pathogenic *Escherichia coli* Is a Virulence Factor That Regulates Outer Membrane Vesicle Biogenesis.” Journal of Infectious Diseases 213, no. 5: 856–865. 10.1093/infdis/jiv506. 26494774

[jev270150-bib-0126] Nairn, C. A. , J. A. Cole , P. V. Patel , N. J. Parsons , J. E. Fox , and H. Smith . 1988. “Cytidine 5'‐Monophospho‐N‐Acetylneuraminic Acid or a Related Compound Is the Low Mr Factor From Human Red Blood Cells Which Induces Gonococcal Resistance to Killing by Human Serum.” Journal of General Microbiology 134, no. 12: 3295–3306. 10.1099/00221287-134-12-3295. 3151997

[jev270150-bib-0127] Natale, P. , T. Brüser , and A. J. M. Driessen . 2008. “Sec‐ and Tat‐Mediated Protein Secretion Across the Bacterial Cytoplasmic Membrane—Distinct Translocases and Mechanisms.” Biochimica et Biophysica Acta 1778, no. 9: 1735–1756. 10.1016/j.bbamem.2007.07.015. 17935691

[jev270150-bib-0128] Nicchi, S. , M. Giuliani , F. Giusti , et al. 2021. “Decorating the Surface of *Escherichia coli* With Bacterial Lipoproteins: A Comparative Analysis of Different Display Systems.” Microbial Cell Factories 20, no. 1: 33. 10.1186/s12934-021-01528-z. 33531008 PMC7853708

[jev270150-bib-0129] Ohara, M. , H. C. Wu , K. Sankaran , and P. D. Rick . 1999. “Identification and Characterization of a New Lipoprotein, NlpI, in *Escherichia coli* K‐12.” Journal of Bacteriology 181, no. 14: 4318–4325. 10.1128/JB.181.14.4318-4325.1999. 10400590 PMC93934

[jev270150-bib-0130] Ojima, Y. , T. Sawabe , K. Konami , and M. Azuma . 2020. “Construction of Hypervesiculation *Escherichia coli* Strains and Application for Secretory Protein Production.” Biotechnology and Bioengineering 117, no. 3: 701–709. 10.1002/bit.27239. 31788781

[jev270150-bib-0131] Ojima, Y. , T. Sawabe , M. Nakagawa , Y. O. Tahara , M. Miyata , and M. Azuma . 2021. “Aberrant Membrane Structures in Hypervesiculating *Escherichia coli* Strain ΔmlaEΔnlpI Visualized by Electron Microscopy.” Frontiers in Microbiology 12: 706525. https://www.frontiersin.org/articles/10.3389/fmicb.2021.706525. 34456889 10.3389/fmicb.2021.706525 PMC8386018

[jev270150-bib-0132] O'Neil . 2016. Tackling Drug‐Resistant Infections Globally Final Report and Recommendations. Consulté 9 avril 2025, à l'adresse. https://amr‐review.org/sites/default/files/160518_Final%20paper_with%20cover.pdf.

[jev270150-bib-0133] Pellegrini, C. , L. Antonioli , G. Lopez‐Castejon , C. Blandizzi , and M. Fornai . 2017. “Canonical and Non‐Canonical Activation of NLRP3 Inflammasome at the Crossroad Between Immune Tolerance and Intestinal Inflammation.” Frontiers in Immunology 8: 36. 10.3389/fimmu.2017.00036. 28179906 PMC5263152

[jev270150-bib-0134] Pérez‐Cruz, C. , M.‐A. Cañas , R. Giménez , et al. 2016. “Membrane Vesicles Released by a Hypervesiculating *Escherichia coli* Nissle 1917 tolR Mutant Are Highly Heterogeneous and Show Reduced Capacity for Epithelial Cell Interaction and Entry.” PLOS ONE 11, no. 12: e0169186. 10.1371/journal.pone.0169186. 28036403 PMC5201253

[jev270150-bib-0135] Pérez‐Cruz, C. , O. Carrión , L. Delgado , G. Martinez , C. López‐Iglesias , and E. Mercade . 2013. “New Type of Outer Membrane Vesicle Produced by the Gram‐Negative Bacterium Shewanella Vesiculosa M7T: Implications for DNA Content.” Applied and Environmental Microbiology 79, no. 6: 1874–1881. 10.1128/AEM.03657-12. 23315742 PMC3592255

[jev270150-bib-0136] Pérez‐Cruz, C. , L. Delgado , C. López‐Iglesias , and E. Mercade . 2015. “Outer‐Inner Membrane Vesicles Naturally Secreted by Gram‐Negative Pathogenic Bacteria.” PLOS ONE 10, no. 1: e0116896. 10.1371/journal.pone.0116896. 25581302 PMC4291224

[jev270150-bib-0137] Pichon, C. , and P. Midoux . 2013. “Mannosylated and Histidylated LPR Technology for Vaccination With Tumor Antigen mRNA.” Methods in Molecular Biology (Clifton, N.J.) 969: 247–274. 10.1007/978-1-62703-260-5_16. 23296939

[jev270150-bib-0138] Prados‐Rosales, R. , B. C. Weinrick , D. G. Piqué , W. R. Jacobs , A. Casadevall , and G. M. Rodriguez . 2014. “Role for Mycobacterium Tuberculosis Membrane Vesicles in Iron Acquisition.” Journal of Bacteriology 196, no. 6: 1250–1256. 10.1128/JB.01090-13. 24415729 PMC3957709

[jev270150-bib-0139] Preston, A. , R. E. Mandrell , B. W. Gibson , and M. A. Apicella . 1996. “The Lipooligosaccharides of Pathogenic Gram‐Negative Bacteria.” Critical Reviews in Microbiology 22, no. 3: 139–180. 10.3109/10408419609106458. 8894399

[jev270150-bib-0140] Pschunder, B. , L. Locati , O. López , et al. 2024. “Outer Membrane Vesicles Derived From Bordetella Pertussis Are Potent Adjuvant That Drive Th1‐Biased Response.” Frontiers in Immunology 15: 1387534. 10.3389/fimmu.2024.1387534. 38650936 PMC11033331

[jev270150-bib-0141] Qing, G. , N. Gong , X. Chen , et al. 2019. “Natural and Engineered Bacterial Outer Membrane Vesicles.” Biophysics Reports 5, no. 4: 184–198. 10.1007/s41048-019-00095-6.

[jev270150-bib-0142] Raeven, R. H. M. , D. Rockx‐Brouwer , G. Kanojia , et al. 2020. “Intranasal Immunization With Outer Membrane Vesicle Pertussis Vaccine Confers Broad Protection Through Mucosal IgA and Th17 Responses.” Scientific Reports 10, no. 1: 7396. 10.1038/s41598-020-63998-2. 32355188 PMC7192948

[jev270150-bib-0143] Rappazzo, C. G. , H. C. Watkins , C. M. Guarino , et al. 2016. “Recombinant M2e Outer Membrane Vesicle Vaccines Protect Against Lethal Influenza A Challenge in BALB/c Mice.” Vaccine 34, no. 10: 1252–1258. 10.1016/j.vaccine.2016.01.028. 26827663

[jev270150-bib-0144] Reimer, S. L. , D. R. Beniac , S. L. Hiebert , et al. 2021. “Comparative Analysis of Outer Membrane Vesicle Isolation Methods With an *Escherichia coli* tolA Mutant Reveals a Hypervesiculating Phenotype With Outer‐Inner Membrane Vesicle Content.” Frontiers in Microbiology 12: 628801. https://www.frontiersin.org/articles/10.3389/fmicb.2021.628801. 33746922 10.3389/fmicb.2021.628801 PMC7973035

[jev270150-bib-0145] Rezaei Adriani, R. , S. L. Mousavi Gargari , H. Bakherad , and J. Amani . 2023. “Anti‐EGFR Bioengineered Bacterial Outer Membrane Vesicles as Targeted Immunotherapy Candidate in Triple‐Negative Breast Tumor Murine Model.” Scientific Reports 13, no. 1: 16403. 10.1038/s41598-023-43762-y. 37775519 PMC10541432

[jev270150-bib-0146] Roden, J. A. , D. H. Wells , B. B. Chomel , R. W. Kasten , and J. E. Koehler . 2012. “Hemin Binding Protein C Is Found in Outer Membrane Vesicles and Protects Bartonella Henselae Against Toxic Concentrations of Hemin.” Infection and Immunity 80, no. 3: 929–942. 10.1128/IAI.05769-11. 22232189 PMC3294634

[jev270150-bib-0147] Roier, S. , F. G. Zingl , F. Cakar , et al. 2016. “A Novel Mechanism for the Biogenesis of Outer Membrane Vesicles in Gram‐Negative Bacteria.” Nature Communications 7, no. 1: 10515. 10.1038/ncomms10515. PMC4737802 26806181

[jev270150-bib-0148] Rojas‐Lopez, M. , M. Martinelli , V. Brandi , et al. 2019. “Identification of Lipid A Deacylase as a Novel, Highly Conserved and Protective Antigen Against Enterohemorrhagic *Escherichia coli* .” Scientific Reports 9, no. 1: 17014. 10.1038/s41598-019-53197-z. 31745113 PMC6863877

[jev270150-bib-0149] Rudi, E. , E. Gaillard , D. Bottero , T. Ebensen , C. A. Guzman , and D. Hozbor . 2024. “Mucosal Vaccination With Outer Membrane Vesicles Derived From Bordetella Pertussis Reduces Nasal Bacterial Colonization After Experimental Infection.” Frontiers in Immunology 15: 1506638. 10.3389/fimmu.2024.1506638. 39669568 PMC11635837

[jev270150-bib-0150] Santos, S. , L. J. d. Arauz , J. Baruque‐Ramos , et al. 2012. “Outer Membrane Vesicles (OMV) Production of *Neisseria meningitidis* Serogroup B in Batch Process.” Vaccine 30, no. 42: 6064–6069. 10.1016/j.vaccine.2012.07.052. 22867717

[jev270150-bib-0151] Schetters, S. T. T. , W. S. P. Jong , S. K. Horrevorts , et al. 2019. “Outer Membrane Vesicles Engineered to Express Membrane‐Bound Antigen Program Dendritic Cells for Cross‐Presentation to CD8+ T Cells.” Acta Biomaterialia 91: 248–257. 10.1016/j.actbio.2019.04.033. 31003032

[jev270150-bib-0152] Schroeder, J. , and T. Aebischer . 2009. “Recombinant Outer Membrane Vesicles to Augment Antigen‐Specific Live Vaccine Responses.” Vaccine 27, no. 48: 6748–6754. 10.1016/j.vaccine.2009.08.106. 19748581

[jev270150-bib-0153] Schwechheimer, C. , and M. J. Kuehn . 2015. “Outer‐Membrane Vesicles From Gram‐Negative Bacteria: Biogenesis and Functions.” Nature Reviews Microbiology 13, no. 10: 605–619. 10.1038/nrmicro3525. 26373371 PMC5308417

[jev270150-bib-0154] Schwechheimer, C. , A. Kulp , and M. J. Kuehn . 2014. “Modulation of Bacterial Outer Membrane Vesicle Production by Envelope Structure and Content.” BMC Microbiology 14, no. 1: 324. 10.1186/s12866-014-0324-1. 25528573 PMC4302634

[jev270150-bib-0155] Schwechheimer, C. , D. L. Rodriguez , and M. J. Kuehn . 2015. “NlpI‐Mediated Modulation of Outer Membrane Vesicle Production Through Peptidoglycan Dynamics in *Escherichia coli* .” MicrobiologyOpen 4, no. 3: 375–389. 10.1002/mbo3.244. 25755088 PMC4475382

[jev270150-bib-0156] Schwechheimer, C. , C. J. Sullivan , and M. J. Kuehn . 2013. “Envelope Control of Outer Membrane Vesicle Production in Gram‐Negative Bacteria.” Biochemistry 52, no. 18: 3031–3040. 10.1021/bi400164t. 23521754 PMC3731998

[jev270150-bib-0157] Scorza, F. B. , A. M. Colucci , L. Maggiore , et al. 2012. “High Yield Production Process for Shigella Outer Membrane Particles.” PLOS ONE 7, no. 6: e35616. 10.1371/journal.pone.0035616. 22701551 PMC3368891

[jev270150-bib-0158] Serruto, D. , M. J. Bottomley , S. Ram , M. M. Giuliani , and R. Rappuoli . 2012. “The New Multicomponent Vaccine Against Meningococcal Serogroup B, 4CMenB : Immunological, Functional and Structural Characterization of the Antigens.” Vaccine 30, no. Suppl 2: B87–97. 10.1016/j.vaccine.2012.01.033. 22607904 PMC3360877

[jev270150-bib-0159] Sinha, R. , H. Koley , D. Nag , S. Mitra , A. K. Mukhopadhyay , and B. Chattopadhyay . 2015. “Pentavalent Outer Membrane Vesicles of *Vibrio cholerae* Induce Adaptive Immune Response and Protective Efficacy in both Adult and Passive Suckling Mice Models.” Microbes and Infection 17, no. 3: 215–227. 10.1016/j.micinf.2014.10.011. 25461799

[jev270150-bib-0160] Sirisaengtaksin, N. , E. J. O'Donoghue , S. Jabbari , A. J. Roe , and A. M. Krachler . 2023. “Bacterial Outer Membrane Vesicles Provide an Alternative Pathway for Trafficking of *Escherichia coli* O157 Type III Secreted Effectors to Epithelial Cells.” mSphere. 8, no. 6: e00520‐23. 10.1128/msphere.00520-23. 37929984 PMC10732017

[jev270150-bib-0161] Song, T. , F. Mika , B. Lindmark , et al. 2008. “A New *Vibrio cholerae* sRNA Modulates Colonization and Affects Release of Outer Membrane Vesicles.” Molecular Microbiology 70, no. 1: 100–111. 10.1111/j.1365-2958.2008.06392.x. 18681937 PMC2628432

[jev270150-bib-0162] Sonntag, I. , H. Schwarz , Y. Hirota , and U. Henning . 1978. “Cell Envelope and Shape of *Escherichia coli*: Multiple Mutants Missing the Outer Membrane Lipoprotein and Other Major Outer Membrane Proteins.” Journal of Bacteriology 136, no. 1: 280–285. 10.1128/jb.136.1.280-285.1978. 361695 PMC218658

[jev270150-bib-0163] Stathopoulos, C. , G. Georgiou , and C. F. Earhart . 1996. “Characterization of *Escherichia coli* Expressing an Lpp'OmpA(46‐159)‐PhoA Fusion Protein Localized in the Outer Membrane.” Applied Microbiology and Biotechnology 45, no. 1–2: 112–119. 10.1007/s002530050657. 8920186

[jev270150-bib-0164] Studier, F. W. , and B. A. Moffatt . 1986. “Use of Bacteriophage T7 RNA Polymerase to Direct Selective High‐level Expression of Cloned Genes.” Journal of Molecular Biology 189, no. 1: 113–130. 10.1016/0022-2836(86)90385-2. 3537305

[jev270150-bib-0165] Sun, J. , X. Lin , Y. He , B. Zhang , N. Zhou , and J. Huang . 2023. “A Bacterial Outer Membrane Vesicle‐Based Click Vaccine Elicits Potent Immune Response Against *Staphylococcus aureus* in Mice.” Frontiers in Immunology 14: 1088501. 10.3389/fimmu.2023.1088501. 36742310 PMC9892643

[jev270150-bib-0166] Sundaram, B. , R. E. Tweedell , S. P. Kumar , and T.‐D. Kanneganti . 2024. “The NLR Family of Innate Immune and Cell Death Sensors.” Immunity 57, no. 4: 674–699. 10.1016/j.immuni.2024.03.012. 38599165 PMC11112261

[jev270150-bib-0167] Taha, M.‐K. , R. Bekkat‐Berkani , and V. Abitbol . 2023. “Changing Patterns of Invasive Meningococcal Disease and Future Immunization Strategies.” Human Vaccines & Immunotherapeutics 19, no. 1: 2186111. 10.1080/21645515.2023.2186111. 37017273 PMC10101658

[jev270150-bib-0168] Takemori, D. , K. Yoshino , C. Eba , H. Nakano , and Y. Iwasaki . 2012. “Extracellular Production of Phospholipase A2 From Streptomyces Violaceoruber by Recombinant *Escherichia coli* .” Protein Expression and Purification 81, no. 2: 145–150. 10.1016/j.pep.2011.10.002. 22019762

[jev270150-bib-0169] Tashiro, Y. , S. Ichikawa , M. Shimizu , et al. 2010. “Variation of Physiochemical Properties and Cell Association Activity of Membrane Vesicles With Growth Phase in *Pseudomonas aeruginosa* .” Applied and Environmental Microbiology 76, no. 11: 3732–3739. 10.1128/AEM.02794-09. 20382806 PMC2876431

[jev270150-bib-0170] Thapa, H. B. , A. M. Müller , A. Camilli , and S. Schild . 2021. “An Intranasal Vaccine Based on Outer Membrane Vesicles against SARS‐CoV‐2.” Frontiers in Microbiology 12: 752739. 10.3389/fmicb.2021.752739. 34803974 PMC8602898

[jev270150-bib-0171] Thomas, J. D. , R. A. Daniel , J. Errington , and C. Robinson . 2001. “Export of Active Green Fluorescent Protein to the Periplasm by the Twin‐Arginine Translocase (Tat) Pathway in *Escherichia coli* .” Molecular Microbiology 39, no. 1: 47–53. 10.1046/j.1365-2958.2001.02253.x. 11123687

[jev270150-bib-0172] Tizard, I. R. 2021. “Adjuvants and Adjuvanticity.” Vaccines for Veterinarians 75–86.e1. 10.1016/B978-0-323-68299-2.00016-2.

[jev270150-bib-0173] Tokuda, H. , and S.‐I. Matsuyama . 2004. “Sorting of Lipoproteins to the Outer Membrane in *E. coli* .” Biochimica et Biophysica Acta 1693, no. 1: 5–13. 10.1016/j.bbamcr.2004.02.005. 15276320

[jev270150-bib-0174] Tollemar, J. , L. Klingspor , and O. Ringdén . 2001. “Liposomal Amphotericin B (AmBisome) for Fungal Infections in Immunocompromised Adults and Children.” Clinical Microbiology and Infection 7: 68–79. 10.1111/j.1469-0691.2001.tb00012.x. 11525221

[jev270150-bib-0175] Torres‐Vanegas, J. D. , N. Rincon‐Tellez , P. Guzmán‐Sastoque , J. D. Valderrama‐Rincon , J. C. Cruz , and L. H. Reyes . 2024. “Production and Purification of Outer Membrane Vesicles Encapsulating Green Fluorescent Protein From *Escherichia coli*: A Step Towards Scalable OMV Technologies.” Frontiers in Bioengineering and Biotechnology 12: 1436352. 10.3389/fbioe.2024.1436352. 39610937 PMC11602331

[jev270150-bib-0176] Toyofuku, M. , N. Nomura , and L. Eberl . 2019. “Types and Origins of Bacterial Membrane Vesicles.” Nature Reviews Microbiology 17, no. 1: Article 1: 13‐24. 10.1038/s41579-018-0112-2. 30397270

[jev270150-bib-0177] Toyofuku, M. , S. Schild , M. Kaparakis‐Liaskos , and L. Eberl . 2023. “Composition and Functions of Bacterial Membrane Vesicles.” Nature Reviews Microbiology 21, no. 7: 415–430. 10.1038/s41579-023-00875-5. 36932221

[jev270150-bib-0178] Tulkens, J. , O. De Wever , and A. Hendrix . 2020. “Analyzing Bacterial Extracellular Vesicles in Human Body Fluids by Orthogonal Biophysical Separation and Biochemical Characterization.” Nature Protocols 15, no. 1: 40–67. 10.1038/s41596-019-0236-5. 31776460

[jev270150-bib-0179] Turnbull, L. , M. Toyofuku , A. L. Hynen , et al. 2016. “Explosive Cell Lysis as a Mechanism for the Biogenesis of Bacterial Membrane Vesicles and Biofilms.” Nature Communications 7, no. 1: 11220. 10.1038/ncomms11220. PMC4834629 27075392

[jev270150-bib-0180] Turner, L. , J. Praszkier , M. L. Hutton , et al. 2015. “Increased Outer Membrane Vesicle Formation in a *Helicobacter pylori* tolB Mutant.” Helicobacter 20, no. 4: 269–283. 10.1111/hel.12196. 25669590

[jev270150-bib-0181] van der Ley, P. A. , A. Zariri , E. van Riet , D. Oosterhoff , and C. P. Kruiswijk . 2021. “An Intranasal OMV‐Based Vaccine Induces High Mucosal and Systemic Protecting Immunity Against a SARS‐CoV‐2 Infection.” Frontiers in Immunology 12: 781280. 10.3389/fimmu.2021.781280. 34987509 PMC8721663

[jev270150-bib-0182] van der Pol, L. , M. Stork , and P. van der Ley . 2015. “Outer Membrane Vesicles as Platform Vaccine Technology.” Biotechnology Journal 10, no. 11: 1689–1706. 10.1002/biot.201400395. 26912077 PMC4768646

[jev270150-bib-0183] van de Waterbeemd, B. , M. Streefland , P. van der Ley , et al. 2010. “Improved OMV Vaccine Against *Neisseria meningitidis* Using Genetically Engineered Strains and a Detergent‐Free Purification Process.” Vaccine 28, no. 30: 4810–4816. 10.1016/j.vaccine.2010.04.082. 20483197

[jev270150-bib-0184] Villena, R. , M. A. P. Safadi , M. T. Valenzuela , J. P. Torres , A. Finn , and M. O'Ryan . 2018. “Global Epidemiology of Serogroup B Meningococcal Disease and Opportunities for Prevention With Novel Recombinant Protein Vaccines.” Human Vaccines & Immunotherapeutics 14, no. 5: 1042–1057. 10.1080/21645515.2018.1458175. 29667483 PMC5989912

[jev270150-bib-0185] Wai, S. N. , B. Lindmark , T. Söderblom , et al. 2003. “Vesicle‐Mediated Export and Assembly of Pore‐Forming Oligomers of the Enterobacterial ClyA Cytotoxin.” Cell 115, no. 1: 25–35. 10.1016/S0092-8674(03)00754-2. 14532000

[jev270150-bib-0186] Wang, H. , K. Liang , Q. Kong , and Q. Liu . 2019. “Immunization With Outer Membrane Vesicles of Avian Pathogenic *Escherichia coli* O78 Induces Protective Immunity in Chickens.” Veterinary Microbiology 236: 108367. 10.1016/j.vetmic.2019.07.019. 31500727

[jev270150-bib-0187] Wang, S. , J. Guo , Y. Bai , et al. 2022. “Bacterial Outer Membrane Vesicles as a Candidate Tumor Vaccine Platform.” Frontiers in Immunology 13: 987419. 10.3389/fimmu.2022.987419. 36159867 PMC9505906

[jev270150-bib-0188] Wang, Y. 2002. “The Function of OmpA in *Escherichia coli* .” Biochemical and Biophysical Research Communications 292, no. 2: 396–401. 10.1006/bbrc.2002.6657. 11906175

[jev270150-bib-0189] Warren, H. S. , C. Fitting , E. Hoff , et al. 2010. “Resilience to Bacterial Infection: Difference Between Species Could Be due to Proteins in Serum.” Journal of Infectious Diseases 201, no. 2: 223–232. 10.1086/649557. 20001600 PMC2798011

[jev270150-bib-0190] Waterbeemd, B. V. D. , G. Zomer , P. Kaaijk , et al. 2013b. “Improved Production Process for Native Outer Membrane Vesicle Vaccine Against *Neisseria meningitidis* .” PLOS ONE 8, no. 5: e65157. 10.1371/journal.pone.0065157. 23741478 PMC3669287

[jev270150-bib-0191] Waterbeemd, B. V. D. , G. Zomer , J. van den IJssel , et al. 2013a. “Cysteine Depletion Causes Oxidative Stress and Triggers Outer Membrane Vesicle Release by *Neisseria meningitidis*; Implications for Vaccine Development.” PLOS ONE 8, no. 1: e54314. 10.1371/journal.pone.0054314. 23372704 PMC3553081

[jev270150-bib-0192] Weyant, K. B. , A. Oloyede , S. Pal , et al. 2023. “A Modular Vaccine Platform Enabled by Decoration of Bacterial Outer Membrane Vesicles With Biotinylated Antigens.” Nature Communications 14, no. 1: 464. 10.1038/s41467-023-36101-2. PMC9883832 36709333

[jev270150-bib-0193] Wo, J. , Z.‐Y. Lv , J.‐N. Sun , H. Tang , N. Qi , and B.‐C. Ye . 2023. “Engineering Probiotic‐Derived Outer Membrane Vesicles as Functional Vaccine Carriers to Enhance Immunity Against SARS‐CoV‐2.” iScience 26, no. 1: 105772. 10.1016/j.isci.2022.105772. 36510593 PMC9729586

[jev270150-bib-0194] Wolf, A. J. , and D. M. Underhill . 2018. “Peptidoglycan Recognition by the Innate Immune System.” Nature Reviews Immunology 18, no. 4: 243–254. 10.1038/nri.2017.136. 29292393

[jev270150-bib-0195] Xian, M. , M. M. Fuerst , Y. Shabalin , and R. N. Reusch . 2007. “Sorting Signal of *Escherichia coli* OmpA Is Modified by Oligo‐(R)‐3‐hydroxybutyrate.” Biochimica et Biophysica Acta 1768, no. 11: 2660–2666. 10.1016/j.bbamem.2007.06.019. 17659252 PMC2266070

[jev270150-bib-0196] Yaguchi, K. , T. Ohgitani , T. Noro , T. Kaneshige , and Y. Shimizu . 2009. “Vaccination of Chickens With Liposomal Inactivated Avian Pathogenic *Escherichia coli* (APEC) Vaccine by Eye Drop or Coarse Spray Administration.” Avian Diseases 53, no. 2: 245–249. 10.1637/8475-092908-Reg.1. 19630231

[jev270150-bib-0197] Yang, J. , F. Jia , Y. Qiao , Z. Hai , and X. Zhou . 2023. “Correlation Between Bacterial Extracellular Vesicles and Antibiotics: A Potentially Antibacterial Strategy.” Microbial Pathogenesis 181: 106167. 10.1016/j.micpath.2023.106167. 37224984

[jev270150-bib-0198] Yethon, J. A. , D. E. Heinrichs , M. A. Monteiro , M. B. Perry , and C. Whitfield . 1998. “Involvement of *waaY*, *waaQ*, and *waaP* in the Modification of *Escherichia coli* Lipopolysaccharide and Their Role in the Formation of a Stable Outer Membrane*.” Journal of Biological Chemistry 273, no. 41: 26310–26316. 10.1074/jbc.273.41.26310. 9756860

[jev270150-bib-0199] Yoon, S. H. , S. K. Kim , and J. F. Kim . 2010. “Secretory Production of Recombinant Proteins in *Escherichia coli* .” Recent Patents on Biotechnology 4, no. 1: 23–29. 10.2174/187220810790069550. 20201800

[jev270150-bib-0200] Zanella, I. , E. König , M. Tomasi , et al. 2021. “Proteome‐Minimized Outer Membrane Vesicles From *Escherichia coli* as a Generalized Vaccine Platform.” Journal of Extracellular Vesicles 10, no. 4: e12066. 10.1002/jev2.12066. 33643549 PMC7886703

[jev270150-bib-0201] Zariri, A. , J. Beskers , B. van de Waterbeemd , et al. 2016. “Meningococcal Outer Membrane Vesicle Composition‐Dependent Activation of the Innate Immune Response.” Infection and Immunity 84, no. 10: 3024–3033. 10.1128/IAI.00635-16. 27481244 PMC5038073

[jev270150-bib-0202] Zavan, L. , N. J. Bitto , E. L. Johnston , D. W. Greening , and M. Kaparakis‐Liaskos . 2019. “ *Helicobacter pylori* Growth Stage Determines the Size, Protein Composition, and Preferential Cargo Packaging of Outer Membrane Vesicles.” Proteomics 19, no. 1‑2: e1800209. 10.1002/pmic.201800209. 30488570

[jev270150-bib-0203] Zhang, W. , J. Lu , S. Zhang , L. Liu , X. Pang , and J. Lv . 2018a. “Development an Effective System to Expression Recombinant Protein in *E. coli* via Comparison and Optimization of Signal Peptides: Expression of Pseudomonas Fluorescens BJ‐10 Thermostable Lipase as Case Study.” Microbial Cell Factories 17, no. 1: 50. 10.1186/s12934-018-0894-y. 29592803 PMC5872382

[jev270150-bib-0204] Zhang, X. , F. Yang , J. Zou , et al. 2018b. “Immunization With *Pseudomonas aeruginosa* Outer Membrane Vesicles Stimulates Protective Immunity in Mice.” Vaccine 36, no. 8: 1047–1054. 10.1016/j.vaccine.2018.01.034. 29406241

[jev270150-bib-0205] Zhao, X. , Y. Fan , D. Wang , et al. 2011. “Immunological Adjuvant Efficacy of Glycyrrhetinic Acid Liposome Against Newcastle Disease Vaccine.” Vaccine 29, no. 52: 9611–9617. 10.1016/j.vaccine.2011.10.053. 22044741

[jev270150-bib-0206] Zhao, X. , Y. Wei , Y. Bu , X. Ren , and Z. Dong . 2025. “Review on Bacterial Outer Membrane Vesicles: Structure, Vesicle Formation, Separation and Biotechnological Applications.” Microbial Cell Factories 24, no. 1: 27. 10.1186/s12934-025-02653-9. 39833809 PMC11749425

[jev270150-bib-0207] Zhu, Z. , F. Antenucci , K. R. Villumsen , and A. M. Bojesen . 2021. “Bacterial Outer Membrane Vesicles as a Versatile Tool in Vaccine Research and the Fight Against Antimicrobial Resistance.” mBio 12, no. 4: e01707‐21. 10.1128/mBio.01707-21. 34372691 PMC8406158

[jev270150-bib-0208] Zhuang, W.‐R. , Y. Wang , W. Nie , et al. 2023. “Bacterial Outer Membrane Vesicle Based Versatile Nanosystem Boosts the Efferocytosis Blockade Triggered Tumor‐Specific Immunity.” Nature Communications 14, no. 1: 1675. 10.1038/s41467-023-37369-0. PMC10039929 36966130

[jev270150-bib-0209] Zingl, F. G. , D. R. Leitner , H. B. Thapa , and S. Schild . 2021. “Outer Membrane Vesicles as Versatile Tools for Therapeutic Approaches.” Microlife 2: uqab006. 10.1093/femsml/uqab006. 37223254 PMC10117751

